# M-Sec facilitates intercellular transmission of HIV-1 through multiple mechanisms

**DOI:** 10.1186/s12977-020-00528-y

**Published:** 2020-07-10

**Authors:** Sameh Lotfi, Hesham Nasser, Osamu Noyori, Masateru Hiyoshi, Hiroaki Takeuchi, Yoshio Koyanagi, Shinya Suzu

**Affiliations:** 1 Division of Infection & Hematopoiesis, Joint Research Center for Human Retrovirus Infection, Kumamoto University, Kumamoto, 860-0811 Japan; 2 International Research Center for Medical Sciences, Kumamoto University, Kumamoto, 860-0811 Japan; 3 Department of Clinical Pathology, Faculty of Medicine, Suez Canal University, Ismailia, 41511 Egypt; 4 Department of Safety Research On Blood and Biological Products, National Institute of Infectious Diseases, Tokyo, 208-0011 Japan; 5 Department of Molecular Virology, Tokyo Medical and Dental University, Tokyo, 113-8519 Japan; 6 Laboratory of Systems Virology, Institute for Frontier Life and Medical Sciences, Kyoto University, Kyoto University, KyotoKyoto, 606-8507 Japan

**Keywords:** HIV-1, M-sec, Tunneling nanotubes, Cell motility

## Abstract

**Background:**

HIV-1 promotes the formation of tunneling nanotubes (TNTs) that connect distant cells, aiding cell-to-cell viral transmission between macrophages. Our recent study suggests that the cellular protein M-Sec plays a role in these processes. However, the timing, mechanism, and to what extent M-Sec contributes to HIV-1 transmission is not fully understood, and the lack of a cell line model that mimics macrophages has hindered in-depth analysis.

**Results:**

We found that HIV-1 increased the number, length and thickness of TNTs in a manner dependent on its pathogenic protein Nef and M-Sec in U87 cells, as observed in macrophages. In addition, we found that M-Sec was required not only for TNT formation but also motility of U87 cells, both of which are beneficial for viral transmission. In fact, M-Sec knockdown in U87 cells led to a significantly delayed viral production in both cellular and extracellular fractions. This inhibition was observed for wild-type virus, but not for a mutant virus lacking Nef, which is known to promote not only TNT formation but also migration of infected macrophages.

**Conclusions:**

By taking advantage of useful features of U87 cells, we provided evidence that M-Sec mediates a rapid and efficient cell–cell transmission of HIV-1 at an early phase of infection by enhancing both TNT formation and cell motility.

## Background

HIV-1 exploits the cell-to-cell infection for its transmission, which is thought to be 100–1000 fold more efficient than infection by cell-free viruses [1, 2]. To date, two models of cell–cell infection of HIV-1 have been reported, namely, virological synapses (VS) [3–5] and tunneling nanotubes (TNTs) [6–9]. The formation of VS is initiated by a direct cell–cell contact, and the viral proteins including Gag and Env localize at the contact site [3, 5]. TNTs, the F-actin-containing plasma membrane extensions, can connect distant cells because they are often several times longer than the diameter of the cell forming them [10–12]. These cell–cell infection routes allow for efficient and rapid viral transmission through reduction of virion exposure to antiviral factors/drugs, neutralizing antibodies, and cytotoxic T lymphocytes.

HIV-1 not only exploits TNTs for its transmission, but also promotes the formation of both long and short TNTs in monocyte-derived macrophages [7–9]. The time course of this process is correlated with that of viral production [13]. TNTs in HIV-1-infected macrophages may contribute not only to viral transmission but also to suppression of antiviral immunity, since they mediate the transfer of viruses and the viral pathogenic protein Nef from infected macrophages to bystander B cells, ultimately resulting in suppression of viral specific IgG2 and IgA production [14]. This finding may explain why antibody responses are ineffective in the majority of HIV-1-infected individuals [15]. It is well known that HTLV-1, another human retrovirus, also promotes TNT formation in CD4^+^ T cells [16, 17]. Furthermore, it has been recently shown that influenza and herpes viruses promote TNTs in their target cells [18–20]. These findings highlight a role of TNTs for viral transmission and pathogenesis, but the molecular mechanisms through which these viruses increase the number and length of TNTs are not fully understood.

Several distinct classes of cellular proteins involved in TNT formation, such as M-Sec [21, 22], LST1 [23], and Myo10 [24], have been identified. We recently discovered a small chemical inhibitor of M-Sec (also known as TNFAIP2) and demonstrated that M-Sec is required for HIV-1 to promote TNT formation in macrophages [25]. M-Sec is a cytosolic protein expressed in cells of monocytic lineage, including macrophages, and plays a critical role in inducing plasma membrane protrusions during TNT biogenesis [21, 22]. When added to the culture of HIV-1-infected macrophages, the inhibitor of M-Sec significantly reduced both TNT formation and HIV-1 production [25]. In contrast, a reduction was not observed with Nef-deficient mutant viruses that fail to promote TNT formation and replicate less efficiently than wild-type viruses [25]. These results suggest that TNTs are important for the transmission of HIV-1 and that M-Sec as a promising target for counteracting HIV-1. However, it remains unclear when and to what extent M-Sec-mediated TNTs contribute to HIV-1 transmission during the course of infection. It is also unknown whether TNT formation is the sole mechanism through which M-Sec facilitates HIV-1 transmission. Due to variations in susceptibility of monocyte-derived macrophages to HIV-1 among donors [26] and the lack of a cell line model that mimics Nef-M-Sec axis-dependent TNT formation in macrophages, in-depth analyses have been hindered.

Here, we report a cell system that precisely phenocopies macrophages in terms of HIV-1-induced TNT formation. Using this convenient system, we also provide evidence that M-Sec enhances not only TNT formation, but also cell motility, thereby facilitating a rapid and efficient cell–cell transmission of HIV-1 in the early phase of infection.

## Results

### U87 cells phenocopy macrophages in terms of HIV-1-induced TNT formation

**Fig. 1:**
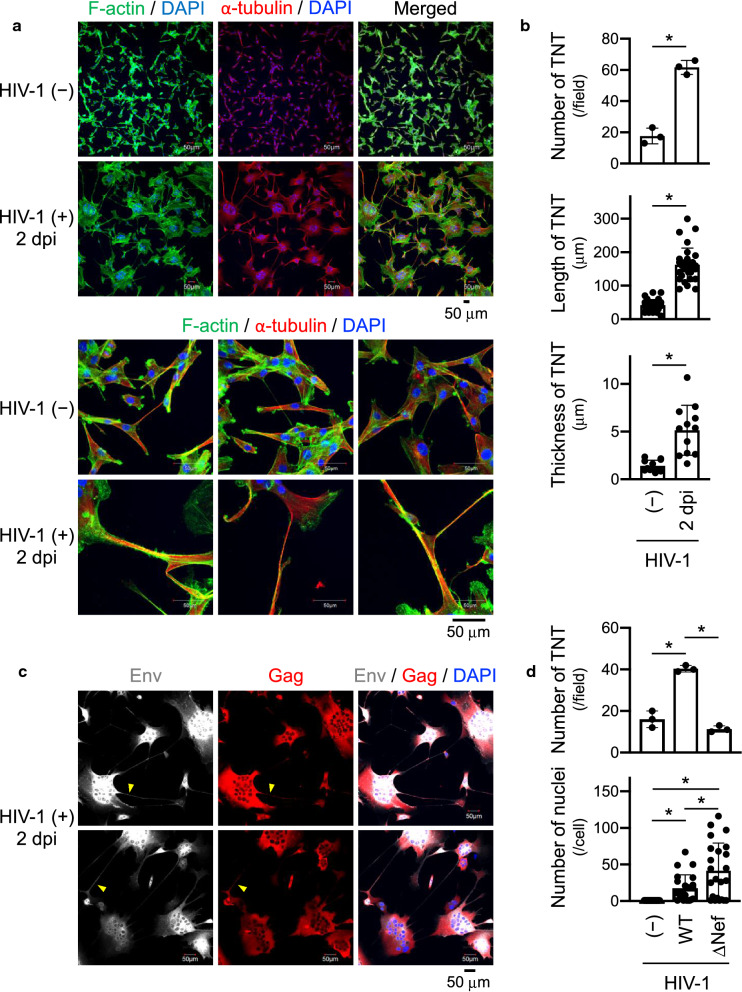
Effect of HIV-1 infection on TNT formation in U87 cells**. a** U87.CD4.CCR5 cells were left uninfected or infected with JRFL (input: 100 ng/ml p24 Gag), cultured for 2 days, and analyzed for F-actin (green) and α-tubulin (red) by immunofluorescence. Nuclei were also stained with DAPI (blue). Lower and higher magnification images are shown in upper and lower panels, respectively. In the lower panels, three different fields for each group are shown. Scale bar: 50 µm. dpi, days postinfection. **b** Cells were prepared as in (**a**) Three different fields were randomly selected, and the number of TNTs per field (top), and the length (middle) and thickness (bottom) of TNTs were quantified. *p < 0.05. dpi, days postinfection. **c** U87.CD4.CCR5 cells were infected with JRFL (input: 100 ng/ml p24 Gag), cultured for 2 days, and analyzed for Env (grey) and Gag (red). Nuclei were also stained with DAPI (blue). Yellow arrowheads indicate TNTs. Scale bar: 50 µm. dpi, days postinfection. **d** U87.CD4.CCR5 cells were left uninfected, or infected with either the wild-type (WT) or Nef-deficient (ΔNef) JRFL virus (input: 100 ng/ml p24 Gag), cultured for 2 days, and analyzed for F-actin by immunofluorescence. Nuclei were also stained with DAPI (blue). Three different fields were randomly selected, and the number of TNTs per field (upper) and the number of nuclei per cell (lower) were quantified. *p < 0.05

When screening cell lines widely-used as HIV-1 targets, we found that U87MG glioma cells (U87.CD4.CCR5 or U87.CD4.CXCR4) expressing HIV-1 receptor (CD4) and co-receptor (CCR5 or CXCR4) [27] express M-Sec at equivalent levels as monocyte-derived macrophages (Additional file 1: Fig. S1a) and form the F-actin^+^ long plasma membrane extensions that do not adhere to substrate (Additional file 1: Fig. S1b), hallmark features of TNTs [10–12]. Thus, we initially analyzed their TNTs after infection. As in macrophages [25], HIV-1 promoted fusion of U87 cells (Fig. 1a, upper panel) and increased the number of TNT in the cells (Fig. 1a, upper panel, and Fig. 1b, top). HIV-1 also increased the length and thickness of TNTs in the cells (Fig. 1a, lower panel, and Fig. 1b, middle and bottom). These TNTs were positive for α-tubulin (Fig. 1a), and HIV-1 Env and Gag (Fig. 1c and Additional file 1: Fig. S2). Moreover, as in macrophages [25], mutant viruses lacking the pathogenic protein Nef (ΔNef) failed to promote TNTs (Fig. 1d, upper panel), which might not be simply due to the weak replication of ΔNef viruses (see Fig. 5b) as they promoted cell fusion more severely than wild-type viruses (Fig. 1d, lower panel, and Additional file 1: Fig. S3). Thus, the U87 cell system phenocopies macrophages in terms of HIV-1-induced TNT formation, which is suitable and convenient for studying the role of TNTs and M-Sec in HIV-1 infection.

### M-Sec is required for both basal- and HIV-1-promoting TNT formation

**Fig. 2:**
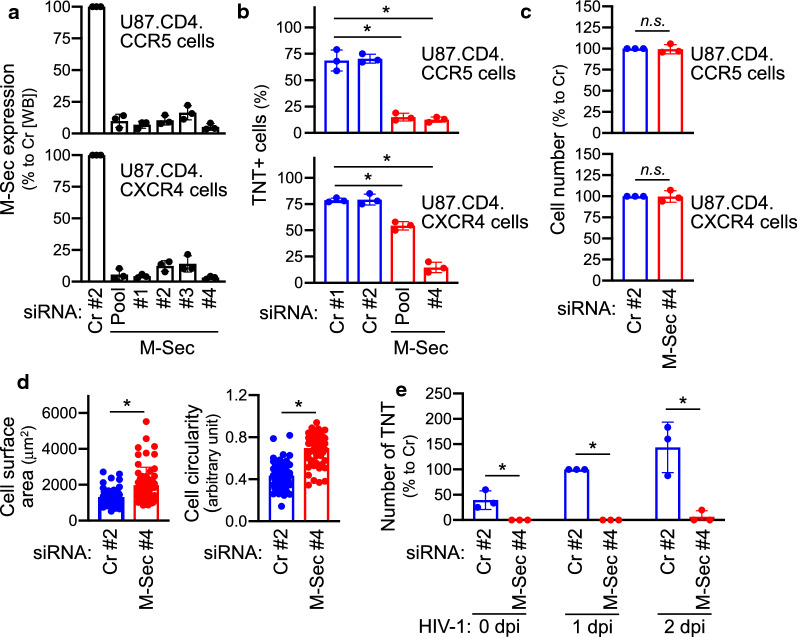
Effect of M-Sec knockdown on TNT formation in U87 cells**. a** U87.CD4.CCR5 (upper) and U87.CD4.CXCR4 cells (lower) were transfected with either control siRNA (Cr pool #2) or M-Sec-specific siRNA (pool, #1, #2, #3, or #4), cultured for 2 days, and analyzed for the expression of M-Sec or actin (as a loading control) by western blotting, followed by densitometric analysis. The band density values are represented as percentages relative to those of the cells transfected with control siRNA (mean ± SD, n = 3). WB, western blotting. **b** U87.CD4.CCR5 (upper) and U87.CD4.CXCR4 cells (lower) were transfected with the indicated siRNA, cultured for 2 days, and analyzed for the percentage of TNT-positive cells in 3 different fields (mean ± SD, n = 3). *p < 0.05. **c** U87.CD4.CCR5 (upper) and U87.CD4.CXCR4 cells (lower) were transfected with either control (Cr pool #2) or M-Sec-specific siRNA (#4) and cultured for 2 days. Cell survival was assessed by the MTT assay. OD values are represented as percentages relative to those of cells transfected with control siRNA (mean ± SD, n = 3). *n.s.*, not significant. **d** U87.CD4.CCR5 cells were transfected with either control (Cr pool #2) or M-Sec-specific siRNA (#4) and cultured for 2 days. The cell surface area (left) and cell circularity (right) were determined using ImageJ 1.52n software. Three different fields were selected, and 20 cells were analyzed in each field (for a total 60 in each group). *p < 0.05. **e** U87.CD4.CCR5 cells were transfected with either control (Cr pool #2) or M-Sec-specific siRNA (#4) and cultured for 2 days. The cells were infected with JRFL (input: 100 ng/ml p24 Gag), cultured for 2 days, and analyzed for TNT number in 3 different fields. The values are represented as percentages relative to those of the control siRNA-transfected cells of 1 dpi (mean ± SD, n = 3). *p < 0.05. *dpi* days postinfection

To test whether basal- and HIV-1-promoting TNT formation in U87 cells depend on M-Sec, we performed knockdown experiments. A mixture (#1 or #2) of four non-targeting siRNAs was used as a control. To knockdown M-Sec, a mixture (Pool) or individual siRNA (#1, #2, #3, or #4) was used. In subsequent experiments, we mainly used M-Sec-targeting siRNA #4 because it was effective in both cells (Fig. 2a and Additional file 1: Fig. S4). M-Sec knockdown reduced basal TNT formation (Fig. 2b and Additional file 1: Fig. S5), which was not due to death of cells (Fig. 2c) but was instead associated with morphological changes evidenced by an increase in the cell surface area and circularity (Fig. 2d and Additional file 1: Fig. S5). The reduced TNT formation by M-Sec knockdown was still observed in HIV-1-infected cells (Fig. 2e and Additional file 1: Fig. S6). Thus, as in macrophages [25], M-Sec is required for HIV-1-promoting TNT formation in U87 cells, confirming that this cell system is suitable for evaluating the role of TNTs and M-Sec in HIV-1 infection.

### M-Sec is also required for cell motility

**Fig. 3:**
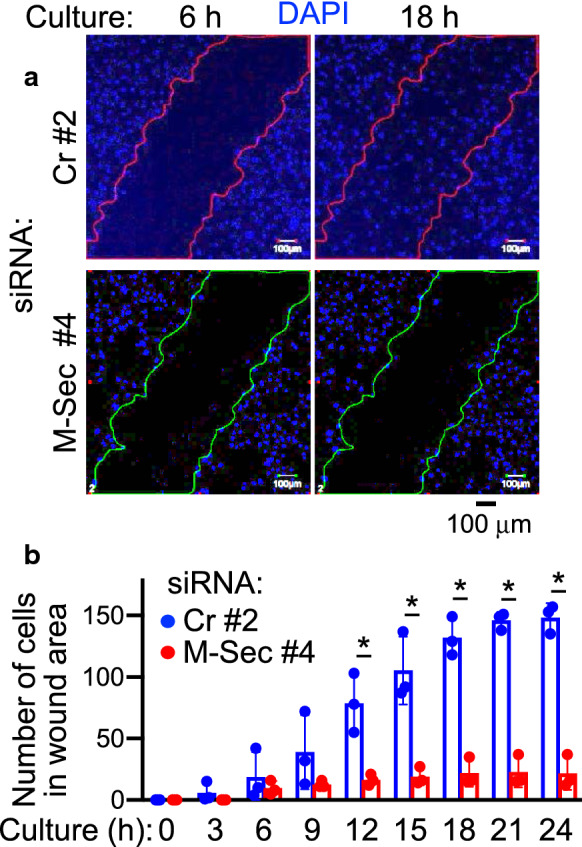
Effect of M-Sec knockdown on wound healing activity of U87 cells**. a**, **b** U87.CD4.CCR5 were transfected with either control (Cr pool #2) or M-Sec-specific siRNA (#4), cultured for 2 days, and analyzed for wound healing activity. In (**a**) typical images are shown (6 or 18 h after assay initialization). Nuclei are shown in blue. Scale bar: 50 µm. In (**b**) cells were cultured for the indicated periods, and cell number in wound area was enumerated in 3 different fields (mean ± SD, n = 3). *p < 0.05

Morphological changes caused by M-Sec knockdown, which include a flattened cell morphology (Fig. 2d), indicate that M-Sec may regulate functions associated with cellular structures other than TNT formation. A recent study demonstrated that transcription factor KLF5 promotes the migration of breast cancer cells partly by upregulating M-Sec [28]. Therefore, we studied the effect of M-Sec on cell motility and found that M-Sec knockdown impaired wound healing activity of U87.CD4.CCR5 cells (Fig. 3) and U87.CD4.CXCR4 cells (Additional file 1: Fig. S7). The migratory activity of U87 cells was also impaired by M-Sec knockdown (Additional file 1: Fig. S8). This phenotype was not specific to U87 cells because we found that M-Sec knockdown in T cell line MT-2 that ectopically expresses M-Sec [25], also significantly reduced cell migratory activity (Additional file 1: Fig. S9). These results suggest that M-Sec is important not only for TNT formation but also for cell motility.

### M-Sec is required for rapid growth of HIV-1 in culture

**Fig. 4:**
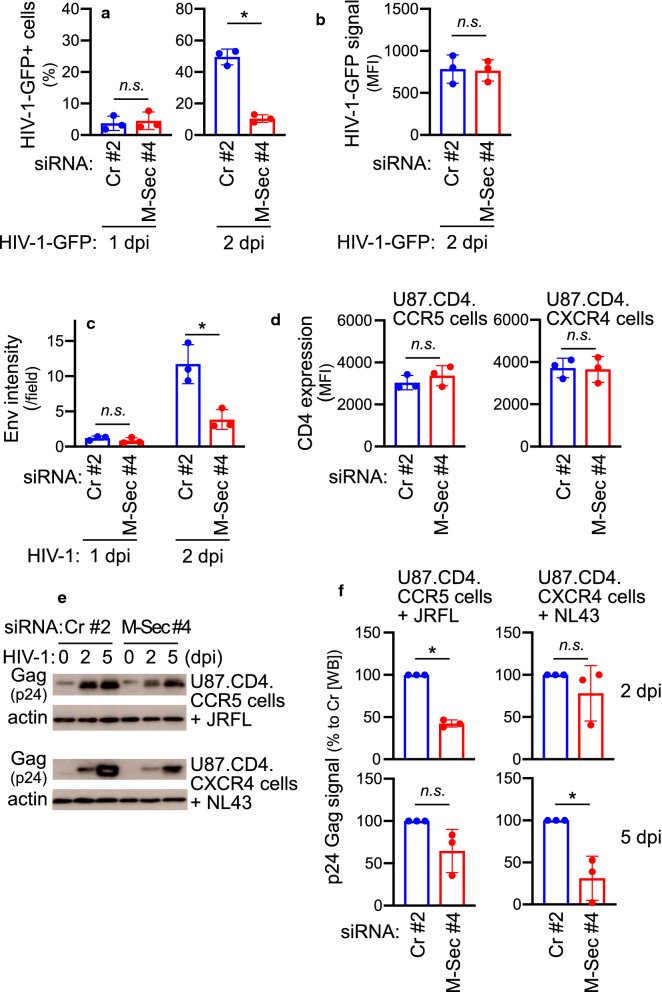
Effect of M-Sec knockdown on quantity of HIV-1 in cellular fraction of U87 cells**. a** U87.CD4.CCR5 cells were transfected with either control (Cr pool #2) or M-Sec-specific siRNA (#4) and cultured for 2 days. Cells were infected with JRFL-GFP (input: 100 ng/ml p24 Gag), cultured for 1–2 days, and analyzed for GFP-positive cell percentage by flow cytometry (mean ± SD, n = 3). *n.s.* not significant. *p < 0.05. dpi, days postinfection. **b** U87.CD4.CCR5 cells were transfected and infected as in (**a**), and cultured for 2 days and analyzed for GFP expression by flow cytometry. Mean fluorescence intensity (MFI) in the GFP-positive fraction is shown (mean ± SD, n = 3). *n.s.* not significant. dpi, days postinfection. **c** U87.CD4.CCR5 cells were transfected with either control (Cr pool #2) or M-Sec-specific siRNA (#4) and cultured for 2 days. Cells were infected with JRFL (input: 100 ng/ml p24 Gag), cultured for 2 days, and analyzed for Env expression by immunofluorescence. The Env signal was quantified in three different fields for each group, and the mean intensity of the Env signal per field is shown. *p < 0.05. *n.s.* not significant; dpi, days postinfection. **d** U87.CD4.CCR5 (left) or U87.CD4.CXCR4 cells (right) were transfected with either control (Cr pool #2) or M-Sec-specific siRNA (#4), cultured for 2 days, and analyzed for CD4 expression by flow cytometry. The mean fluorescence intensity (MFI) of CD4 is shown (mean ± SD, n = 3). *n.s.* not significant. **e** U87.CD4.CCR5 (upper) or U87.CD4.CXCR4 cells (lower) were transfected with either control (Cr pool #2) or M-Sec-specific siRNA (#4), and cultured for 2 days. U87.CD4.CCR5 and U87.CD4.CXCR4 cells were infected with JRFL (input: 100 ng/ml p24 Gag) and NL43 (input: 1 ng/ml p24 Gag), respectively, and cultured for 5 days. Total cell lysates were prepared at day 0, 2, or 5, and subjected to western blotting analysis of p24 Gag or actin (as a loading control). dpi, days postinfection. **f** U87.CD4.CCR5 (left) or U87.CD4.CXCR4 cells (right) were transfected, infected, and analyzed by western blotting as in (**e**). p24 Gag intensity was quantified (day 2 and day 5), and represented as percentage relative to cells transfected with control siRNA (mean ± SD, n = 3). *p < 0.05. *n.s.* not significant, *WB* western blotting; *dpi* days postinfection

**Fig. 5:**
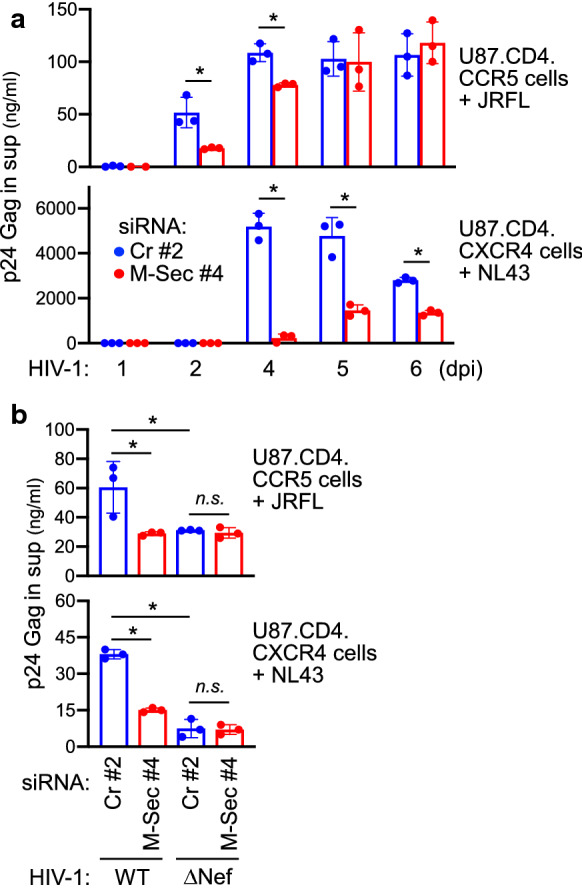
Effect of M-Sec knockdown on the quantity of HIV-1 in culture supernatants of U87 cells**. a** U87.CD4.CCR5 (upper) or U87.CD4.CXCR4 cells (lower) were transfected with either control (Cr pool #2) or M-Sec-specific siRNA (#4), and cultured for 2 days. Next, U87.CD4.CCR5 and U87.CD4.CXCR4 cells were infected with JRFL (input: 100 ng/ml p24 Gag) and NL43 (input: 1 ng/ml p24 Gag), respectively, and cultured for 6 days. The concentration of p24 Gag in culture supernatants (day 0, 1, 2, 4, 5, and 6) was determined by ELISA (mean ± SD, n = 3). *p < 0.05. sup, supernatants; dpi, days postinfection. **b** U87.CD4.CCR5 (upper) or U87.CD4.CXCR4 cells (lower) were transfected with either control (Cr pool #2) or M-Sec-specific siRNA (#4), and cultured for 2 days. Next, U87.CD4.CCR5 cells were infected with the wild-type (WT) or Nef-deficient (ΔNef) JRFL virus (input: 100 ng/ml p24 Gag), and U87.CD4.CXCR4 cells were infected with WT or ΔNef NL43 virus (input: 1 ng/ml p24 Gag). Cells were cultured for 2 days, and analyzed for p24 Gag concentration in supernatants by ELISA (mean ± SD, n = 3). *p < 0.05. *n.s.* not significant, *sup* supernatants

We next sought to answer how and to what extent M-Sec contributes to cell–cell transmission of HIV-1. We first used replication-competent HIV-1 (JRFL strain)-GFP viruses coexpressing Nef and GFP from a bicistronic mRNA in a manner dependent on the HIV-1 LTR promoter (see Methods section). In this system, the expression of viral genes integrated into the cellular genome can be monitored through flow cytometry of GFP. As shown in Fig. 4a, at 2 dpi (right) but not at 1 dpi (left), the percentage of GFP^+^ cells in the M-Sec knockdown culture was lower than that in the control culture. However, GFP signal for individual cells was similar in these two cultures (Fig. 4b), suggesting that M-Sec is not involved in the expression of viral genes, but facilitates cell–cell viral transmission. To confirm this, we monitored the levels of viral proteins, Env and Gag. When assessed by immunofluorescence, Env intensity per field in the M-Sec knockdown culture was lower than that in the control culture at 2 dpi (Fig. 4c, right, and Additional file 1: Fig. S10) but not at 1 dpi (Fig. 4c, left). Moreover, the percentage of Env^+^ cells at 2 dpi in the M-Sec knockdown culture was lower than that in the control culture (Additional file 1: Fig. S11, left), but the Env signal for individual cells was similar in these two cultures (Additional file 1: Fig. S11, right), as we observed for virally expressed GFP (see Fig. 4a). Although M-Sec knockdown did not affect the expression of HIV-1 receptor CD4 (Fig. 4d), Gag expression was also reduced after M-Sec knockdown when assessed by western blotting of the cellular fraction. In U87.CD4.CCR5 cells infected with JRFL strain, the expression of Gag in the M-Sec knockdown culture was lower than that in the control culture at 2 dpi (Fig. 4e, upper panel, and Fig. 4f, left). In U87.CD4.CXCR4 cells infected with NL43 strain, the viral production kinetics of which was different from that of JRFL-infected U87.CD4.CCR5 cells (see Fig. 5a for details), Gag expression in the M-Sec knockdown culture was lower than that in the control culture at 5 dpi (Fig. 4e, lower panel, and Fig. 4f, right).

The viral replication was clearly different between the two cultures (Fig. 5a). First, the overall level of JRFL in U87.CD4.CCR5 cells was comparable to that in macrophages [25] but was much lower than that of NL43 in U87.CD4.CXCR4 cells. Second, the replication of JRFL in U87.CD4.CCR5 cells gradually increased and became stable, whereas that of NL43 in U87.CD4.CXCR4 cells increased sharply and declined gradually. These differences might be due to a different extent of cell fusion and death between the cultures (Additional file 1: Fig. S12): JRFL-infected U87.CD4.CCR5 cells were severely fused but relatively resistant to cell death when compared to NL43-infected U87.CD4.CXCR4 cells. Thus, we analyzed how M-Sec knockdown resulted in viral production in the supernatants in the two culture systems. In JRFL-infected U87.CD4.CCR5 cells, p24 concentration difference between the control and M-Sec knockdown cultures was more pronounced at earlier time points, such as at 2 and 4 dpi, but not at the later time points of 5 and 6 dpi (Fig. 5a, upper panel). The result for the later time points such as 6 dpi may be explained by the formation of giant cells and the increased number of cells over time in the culture (Additional file 1: Fig. S12, left), in which TNTs and cell motility are of minor importance. Meanwhile, in NL43-infected U87.CD4.CXCR4 cells, differences in p24 between the two cultures were detectable even at the later time points (Fig. 5a, lower panel). In both systems, the difference in earlier time points was evident (2 dpi for JRFL-infected U87.CD4.CCR5 cells and 4 dpi for NL43-infected U87.CD4.CXCR4 cells). We confirmed that impaired viral production after transfection of M-Sec siRNA #4 was not due to an off-target effect since other M-Sec siRNAs were also effective (Additional file 1: Fig. S13a). Furthermore, the observed effect was not due to an induction of IFNs because B18R, an inhibitor of type I IFN [29], did not restore the decrease in viral production caused by M-Sec knockdown Additional file 1: Fig. S13b). In addition, there was no statistical difference in the expression of *MX2*, an IFN-stimulated gene, between the two cultures (Additional file 1: Fig. S13c). In fact, M-Sec knockdown reduced production of wild-type (WT) viruses, but not that of Nef-deficient (ΔNef) viruses, which replicate less efficiently than the wild-type viruses (Fig. 5b). This result was consistent with the fact that Nef is essential for enhanced TNT formation (see Fig. 1d).

In summary, our findings strongly suggest that M-Sec mediates a rapid and efficient cell–cell transmission of HIV-1 in an early phase of infection, which is likely due to enhanced TNT formation and cell motility. In fact, the number of HIV-1-infected TNT^+^ U87 cells that repopulated the wound area was low in the M-Sec knockdown culture (Additional file 1: Fig. S14).

## Discussion

In this study, we demonstrated that HIV-1 increases the number, length and thickness of TNTs via Nef and M-Sec in U87 cells, in a manner similar to that observed for macrophages. To the best of our knowledge, U87 cells are the first cell model to precisely mimic macrophages in terms of HIV-1-induced TNT formation. Through knockdown experiments in U87 cells, we further confirmed that M-Sec is a positive regulator of HIV-1 transmission. Moreover, our study raised the possibility that the role of M-Sec in HIV-1 transmission is not only due to TNT-inducing activity but also due to its effect on cell motility.

We utilized several features of U87 cells to highlight the importance of M-Sec in HIV-1 transmission during the early phase of infection. Despite its impact on cell morphology (Fig. 2d), M-Sec knockdown does not appear to affect other steps in HIV-1 life cycle, apart from transmission. Experiments using HIV-1-GFP viruses (Fig. 4a, b) suggest that M-Sec knockdown minimally affects viral entry and expression of viral genes. M-Sec knockdown reduced the amount of both p24 Gag (Fig. 4e, f) and its precursor p41 or p55 (Additional file 1: Fig. S15), suggesting that it did not affect processing of viral proteins. In addition, M-Sec knockdown reduced both cellular- (Fig. 4e, f) and extracellular (Fig. 5a) concentrations of p24 Gag, further suggesting that it did not affect viral budding. We observed that differences in viral production between control and M-Sec knockdown culture diminished over time (Fig. 5a, upper panel), supporting a role for M-Sec in mediating rapid cell–cell viral transmission during the early stages of infection.

We observed a pronounced impairment of wound healing activity of U87 cells after M-Sec knockdown (Fig. 3 and Additional file 1: Fig. S7), as well as reduced migratory activity of both U87 cells (Additional file 1: Fig. S8) and MT-2 cells (Additional file 1: Fig. S9). Because migration of infected cells can increase the likelihood of encountering uninfected cells, M-Sec appears to promote HIV-1 transmission through forming TNTs and enhancing cell motility. It has previously been reported that HIV-1 enhances three-dimensional mesenchymal migration of infected macrophages [30, 31]. This increase in migration depends on Nef [30], which is also critical for promoting TNT formation by HIV-1 (Fig. 1d) [25]. Thus, M-Sec may play a role in enhancing mesenchymal migration of infected macrophages in a Nef-dependent manner, as observed similarly in TNT formation. To test this possibility, further studies of the molecular mechanisms through which M-Sec regulates TNT formation and cell motility are needed, including the involvement of Nef.

Small GTPases may regulate both TNT formation and cell migration [21, 28]. Indeed, M-Sec has been reported to interact with small GTPase Ral in the mouse macrophage-like cell line RAW264.7, which contributes to TNT formation in these cells [21, 22]. However, an inhibitor of Ral (BQU57) [32] did not reduce basal formation of TNTs in U87 cells (Additional file 1: Fig. S16). Likewise, an inhibitor of another small GTPase Cdc42 (ZCL278) [33] did not reveal any inhibitory effect on basal formation of TNTs in the cells (Additional file 1: Fig. S16). Thus, to understand the relative contribution of M-Sec-mediated TNTs and cell motility to HIV-1 transmission, the identification of small GTPase(s) essential for the two distinct functions of M-Sec will be important. The U87 cell system would be useful for future studies investigating this.

M-Sec knockdown reduced the migratory capacity of MT-2 cells (Fig. S9), which are well-known HTLV-1 persistently infected cells. We recently found that M-Sec knockdown also reduced TNT formation in MT-2 cells and transfer of HTLV-1 from infected cells to uninfected Jurkat T cells (will be published elsewhere). These studies suggest that M-Sec plays a role in transmission of both HTLV-1 and HIV-1. M-Sec may be therefore a conserved cellular target for counteracting both viruses.

## Conclusions

In summary, our findings suggest that M-Sec mediates a rapid and efficient cell–cell transmission of HIV-1 during the early stages of infection by enhancing TNT formation and cell motility.

## Methods

### U87 cells

U87.CD4.CCR5 (#4035) and U87.CD4.CXCR4 cells (#4036) were obtained through the NIH AIDS Reagent Program [27] and were maintained in DMEM-10% FCS containing 1 µg/ml puromycin and 300 µg/ml G418.

### RNA interference

Knockdown of M-Sec in U87 cells was performed using Lipofectamine RNAiMAX reagent (Invitrogen) and siRNA (Dharmacon). U87 cells were seeded onto 24-well plates or 4-chamber glass slides, cultured with antibiotic-free media for 1 day, and then, transfected with 10 pmol/well of siRNA using 1.5 μl/well of Lipofectamine RNAiMAX. siRNAs used are as follows: non-targeting siRNAs (pool #1; D-001206-13, and pool #2; D-001206-14), M-Sec-specific siRNAs (pool; M-012267-01, #1; D-012267-01, #2; D-012267-02, #3; D-012267-03, and #4; D-012267-17). After 6 h of transfection, the culture medium was replaced with fresh medium, and cells were cultured for another 2 days.

### TNT counts and image analysis

The number of TNTs, including both short and long TNTs, was quantified according to the criteria described in a previous report [13]. TNTs in U87 cells were readily distinguishable from filopodia on their length (Fig. 1a), and F-actin^+^ protrusions longer than approximately 10 µm were considered to be TNT in this study. When cells were infected with HIV-1, we quantified the number of TNTs per field because cells are fused at different degrees and those fused cells often form multiple TNTs (see Fig. 1a and c). The length and thickness of TNTs and the signal of Env were quantified using ImageJ 1.52n software (NIH). The cell surface area and circularity were also quantified using the same software, according to a recent report [34].

### Wound healing assay

U87 cells were stained with Hoechst 33,342 (Dojindo, Kumamoto, Japan) to visualize nuclei, and a linear wound was generated in the cell sheet using a 200 µl pipette tip. The floating cells were removed by washing with media. Cells were then incubated for 24 h and recorded at 20-min intervals on an FV1200 confocal laser-scanning microscope, and the number of cells in the wound area was enumerated.

### HIV-1 viruses and p24 Gag ELISA

Recombinant HIV-1 viruses were prepared, as described previously [25]. HEK293A cells (Invitrogen) cultured with DMEM-10% FCS were used as virus producing cells. In brief, cells were seeded onto 12-well tissue culture plates and transfected with HIV-1 proviral plasmids using Lipofectamine 2000 reagent (Invitrogen). After 6 h of transfection, the culture medium was replaced with fresh medium, and the cells were cultured for an additional 48 h. The supernatants containing recombinant viruses were clarified by centrifugation, analyzed for their Gag protein concentrations by ELISA (MBL, Nagoya, Japan), and stored at − 70 °C before use. The CCR5-tropic JRFL and its Nef-deficient mutant (a frameshift mutation at the Xho I site of Nef) were used [35]. The CXCR4-tropic NL43 and its Nef-deficient mutant plasmids were provided by A. Adachi (Kansai Medical University, Osaka, Japan) [36]. A GFP-expressing JRFL plasmid, in which a BstBI-XhoI of the GFP-tagged HIV-1 DNA (NL-CSFV3-EGFP) [37] was inserted into the JRFL infectious plasmid, was also used.

### HIV-1 infection

HIV-1 infection was performed as described previously [25]. U87 cells cultured on 24-well tissue culture plates were incubated with 200 µl of 293A supernatants containing HIV-1 (Gag concentration: 100 ng/ml and 1 ng/ml for JRFL and NL43, respectively) for 2 h at 37 °C. JRFL and NL43 were used for U87.CD4.CCR5 cells and U87.CD4.CXCR4 cells, respectively. Next, the cells were washed twice with PBS to remove any unbound viruses and cultured with DMEM-10% FCS. To monitor viral replication, we determined the concentration of p24 Gag protein in culture supernatants by ELISA.

### Immunofluorescence

Immunofluorescence analysis was performed, as described previously [25]. In brief, cells were directly fixed in 4% paraformaldehyde, permeabilized with 0.1% Triton X-100, and stained with phalloidin conjugated to AlexaFluor633 and DAPI (both from Molecular Probes) to visualize F-actin and nuclei, respectively. Cells were also stained with antibodies against α-tubulin (#DM1A; Sigma), Env (#KD247; provided by S. Matsushita, Kumamoto University, Kumamoto, Japan), or Gag (#Kal-1; Dako) for 12 h followed by anti-mouse IgG-AlexaFluor488, anti-human IgG-AlexaFluor488, or anti-mouse IgG-AlexaFluor488, respectively (all from Molecular Probes). Signals were visualized using an FV1200 confocal laser-scanning microscope (Olympus), and image processing was performed using the FV Viewer ver. 4.1 software (Olympus).

### Western blotting

Western blotting was performed as described previously [25]. In brief, U87 cells were lysed on ice with Nonidet P-40 lysis buffer containing protease inhibitors, and total cell lysates were then subjected to western blotting using the following antibodies: anti-M-Sec (#SC-30138; Santa Cruz Biotechnology), anti-Gag (#65005; BioAcademia, Osaka, Japan), and actin (ab8227; Abcam, as a loading control). Next, they were detected using HRP-labeled secondary antibodies (GE Healthcare), the Immunostar LD Western blotting detection reagent (Wako, Osaka, Japan), and an image analyzer (ImageQuant LAS 4000; GE Healthcare). Band density of Gag or M-Sec was quantified using the ImageJ software after normalization to the density of the actin band.

### Cell survival

Viable cell number assessed using MTT reagent [25]. The absorbance of the wells was measured at 595 nm.

### Flow cytometry

U87 cells were detached from the plates using enzyme-free cell dissociation buffer (Life Technologies) and analyzed for cell surface expression of CD4 by flow cytometry on FACSVerse (BD Biosciences) using FlowJo software. Allophycocyanin (APC)-labeled anti-CD4 antibody (#RPA-T4; Biolegend) was used. U87 cells infected with JRFL-GFP viruses were detached, fixed in Fixation buffer (BioLegend), and analyzed for GFP signal by flow cytometry.

### Statistical analysis

Statistical significance of intersample differences was determined using a paired Student *t*-test. Mann–Whitney *U* test was used for comparison of data sets with non-normal distributions using Prism 8 (GraphPad). *P* values < 0.05 were considered significant.

## Supplementary information

**Additional file 1.** Supplemental Fig. S1–Fig. S16.

## Data Availability

Not applicable.

## Abbreviations

TNT: Tunneling nanotube
dpi: Days postinfection
VS: Virological synapses
WT: Wild-type
ΔNef: Nef-deficient
