# Supplementary material for: M-Sec facilitates intercellular transmission of HIV-1 through multiple mechanisms
Source: Retrovirology. 2020 Jul 10;17:20. doi: 10.1186/s12977-020-00528-y (PMC7350586; doi:10.1186/s12977-020-00528-y)
Supplement: Supplementary file 1 — Additional file 1. Supplemental Fig. S1–Fig. S16. [file 12977_2020_528_MOESM1_ESM.pptx]

## Slide 1
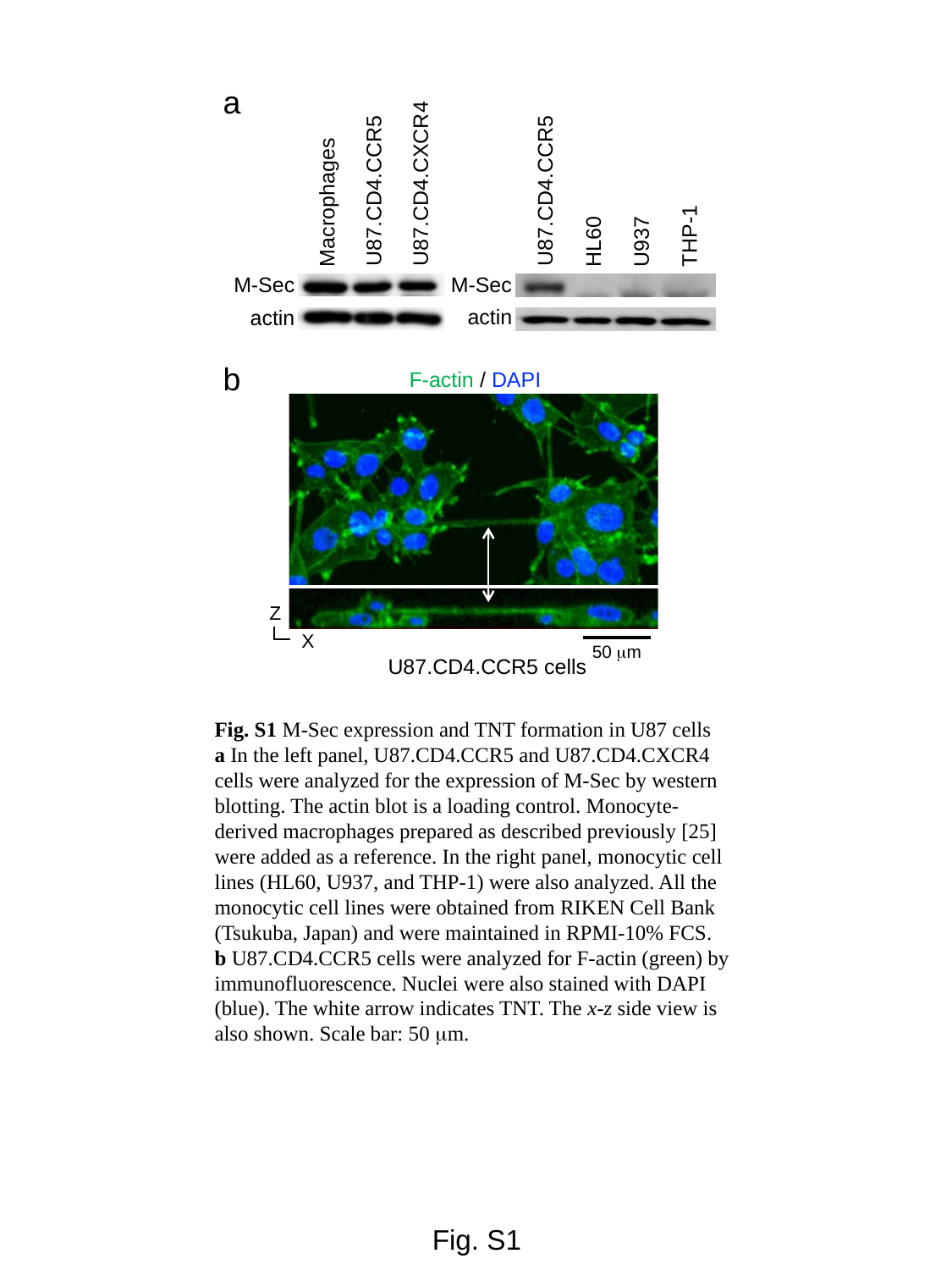

a
U87.CD4.CXCR4
U87.CD4.CCR5
U87.CD4.CCR5
Macrophages
THP-1
HL60
U937
M-Sec
M-Sec
actin
actin
b
F-actin / DAPI
Z
X
50 mm
U87.CD4.CCR5 cells
Fig. S1 M-Sec expression and TNT formation in U87 cells
a In the left panel, U87.CD4.CCR5 and U87.CD4.CXCR4 cells were analyzed for the expression of M-Sec by western blotting. The actin blot is a loading control. Monocyte-derived macrophages prepared as described previously [25] were added as a reference. In the right panel, monocytic cell lines (HL60, U937, and THP-1) were also analyzed. All the monocytic cell lines were obtained from RIKEN Cell Bank (Tsukuba, Japan) and were maintained in RPMI-10% FCS.
b U87.CD4.CCR5 cells were analyzed for F-actin (green) by immunofluorescence. Nuclei were also stained with DAPI (blue). The white arrow indicates TNT. The x-z side view is also shown. Scale bar: 50 mm.
Fig. S1

## Slide 2
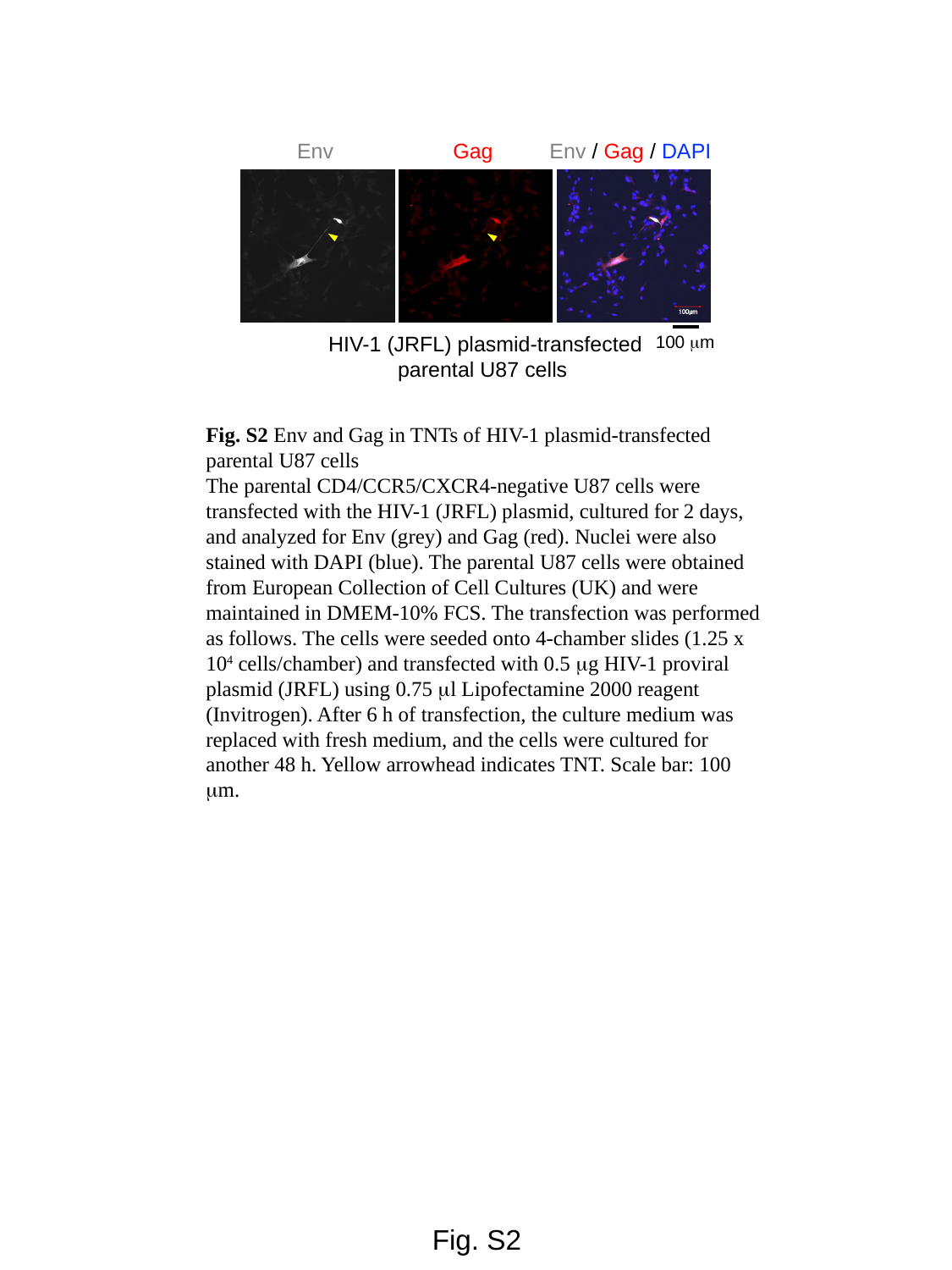

Env
Gag
Env / Gag / DAPI
100 mm
HIV-1 (JRFL) plasmid-transfected
parental U87 cells
Fig. S2 Env and Gag in TNTs of HIV-1 plasmid-transfected parental U87 cells
The parental CD4/CCR5/CXCR4-negative U87 cells were transfected with the HIV-1 (JRFL) plasmid, cultured for 2 days, and analyzed for Env (grey) and Gag (red). Nuclei were also stained with DAPI (blue). The parental U87 cells were obtained from European Collection of Cell Cultures (UK) and were maintained in DMEM-10% FCS. The transfection was performed as follows. The cells were seeded onto 4-chamber slides (1.25 x 10^4^ cells/chamber) and transfected with 0.5 mg HIV-1 proviral plasmid (JRFL) using 0.75 ml Lipofectamine 2000 reagent (Invitrogen). After 6 h of transfection, the culture medium was replaced with fresh medium, and the cells were cultured for another 48 h. Yellow arrowhead indicates TNT. Scale bar: 100 mm.
Fig. S2

## Slide 3
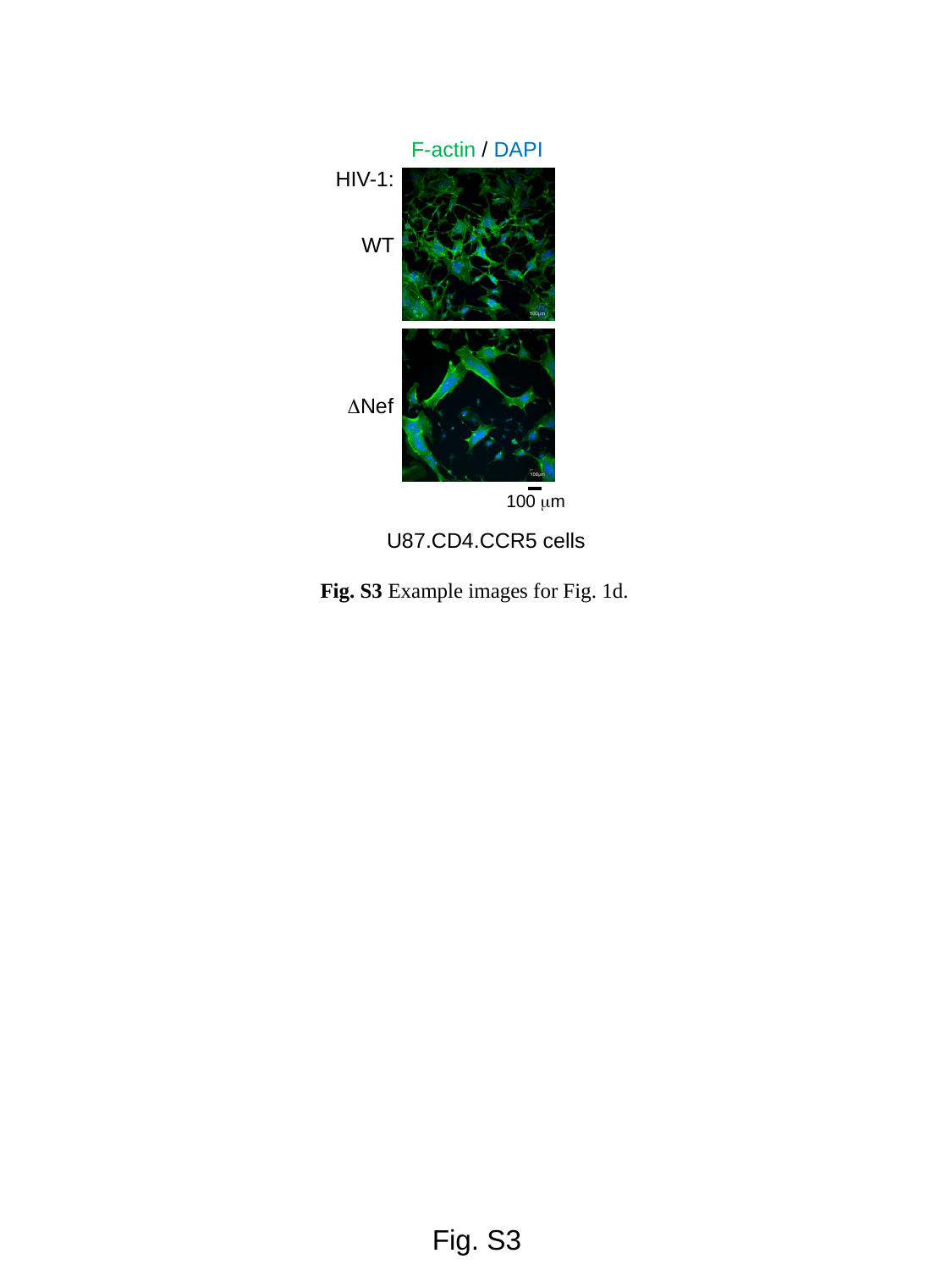

F-actin / DAPI
HIV-1:
WT
DNef
100 mm
U87.CD4.CCR5 cells
Fig. S3 Example images for Fig. 1d.
Fig. S3

## Slide 4
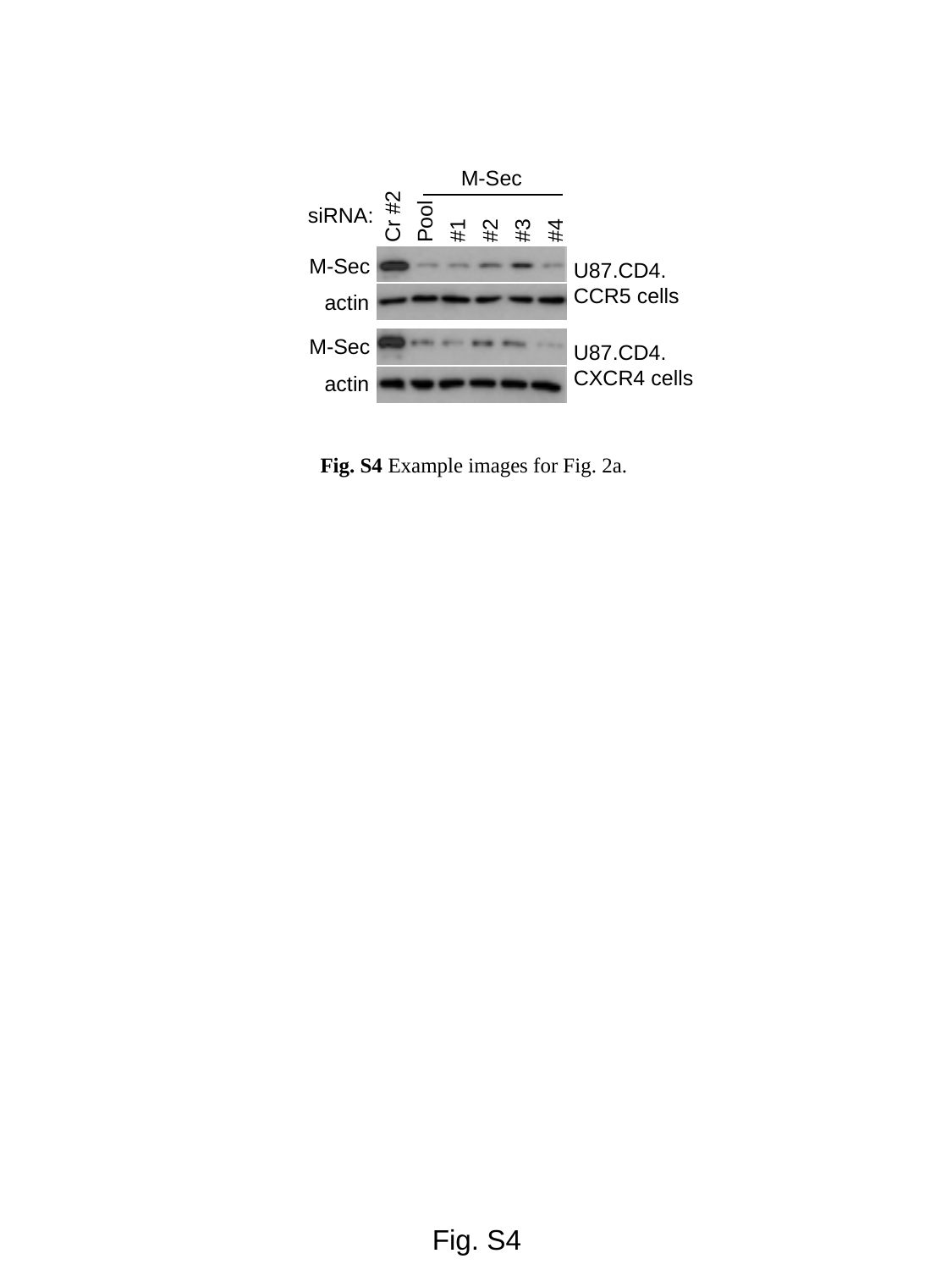

M-Sec
siRNA:
Cr #2
Pool
#1
#2
#3
#4
M-Sec
U87.CD4.
CCR5 cells
actin
M-Sec
U87.CD4.
CXCR4 cells
actin
Fig. S4 Example images for Fig. 2a.
Fig. S4

## Slide 5
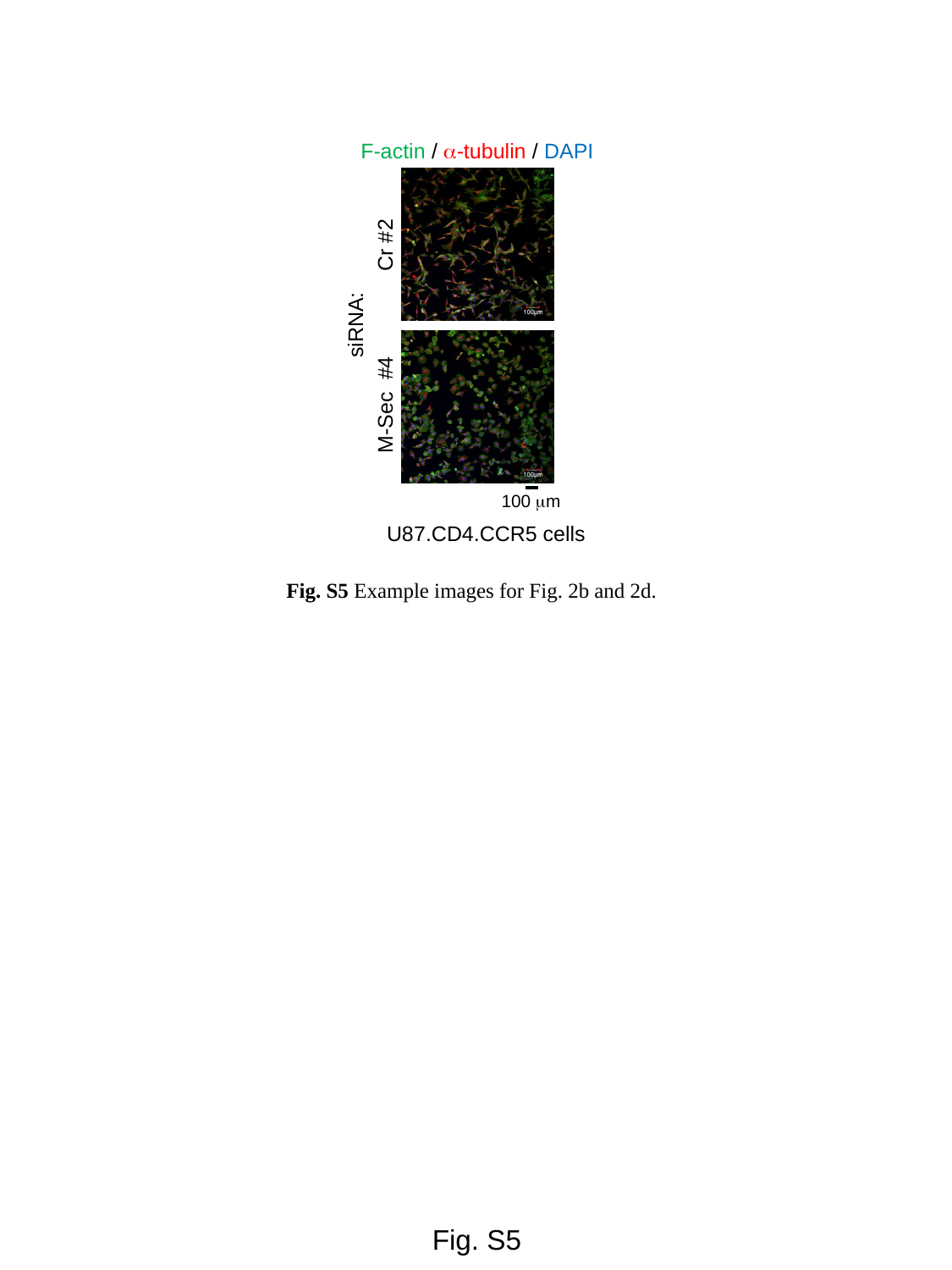

F-actin / a-tubulin / DAPI
Cr #2
siRNA:
M-Sec #4
100 mm
U87.CD4.CCR5 cells
Fig. S5 Example images for Fig. 2b and 2d.
Fig. S5

## Slide 6
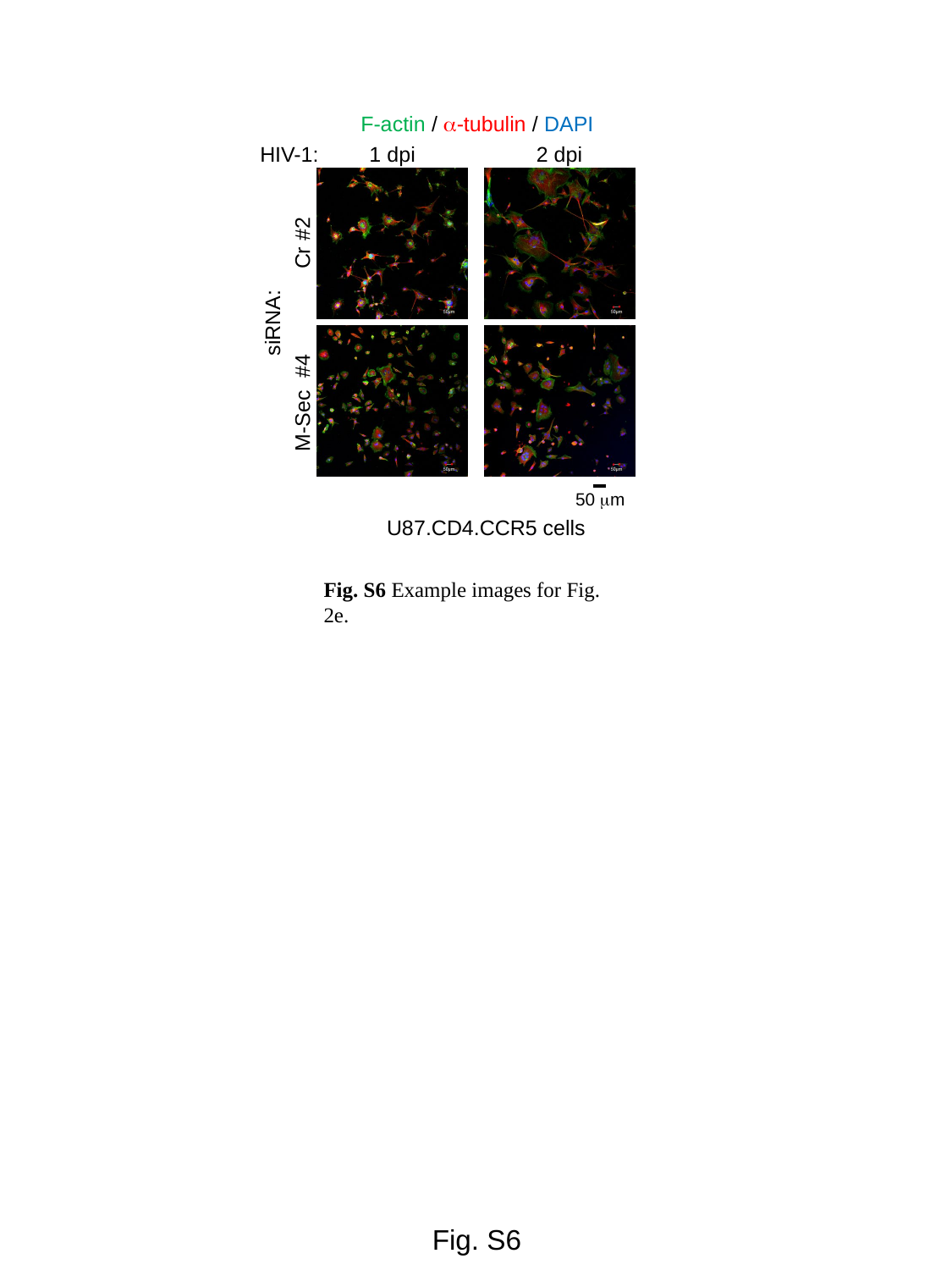

F-actin / a-tubulin / DAPI
HIV-1:
1 dpi
2 dpi
Cr #2
siRNA:
M-Sec #4
50 mm
U87.CD4.CCR5 cells
Fig. S6 Example images for Fig. 2e.
Fig. S6

## Slide 7
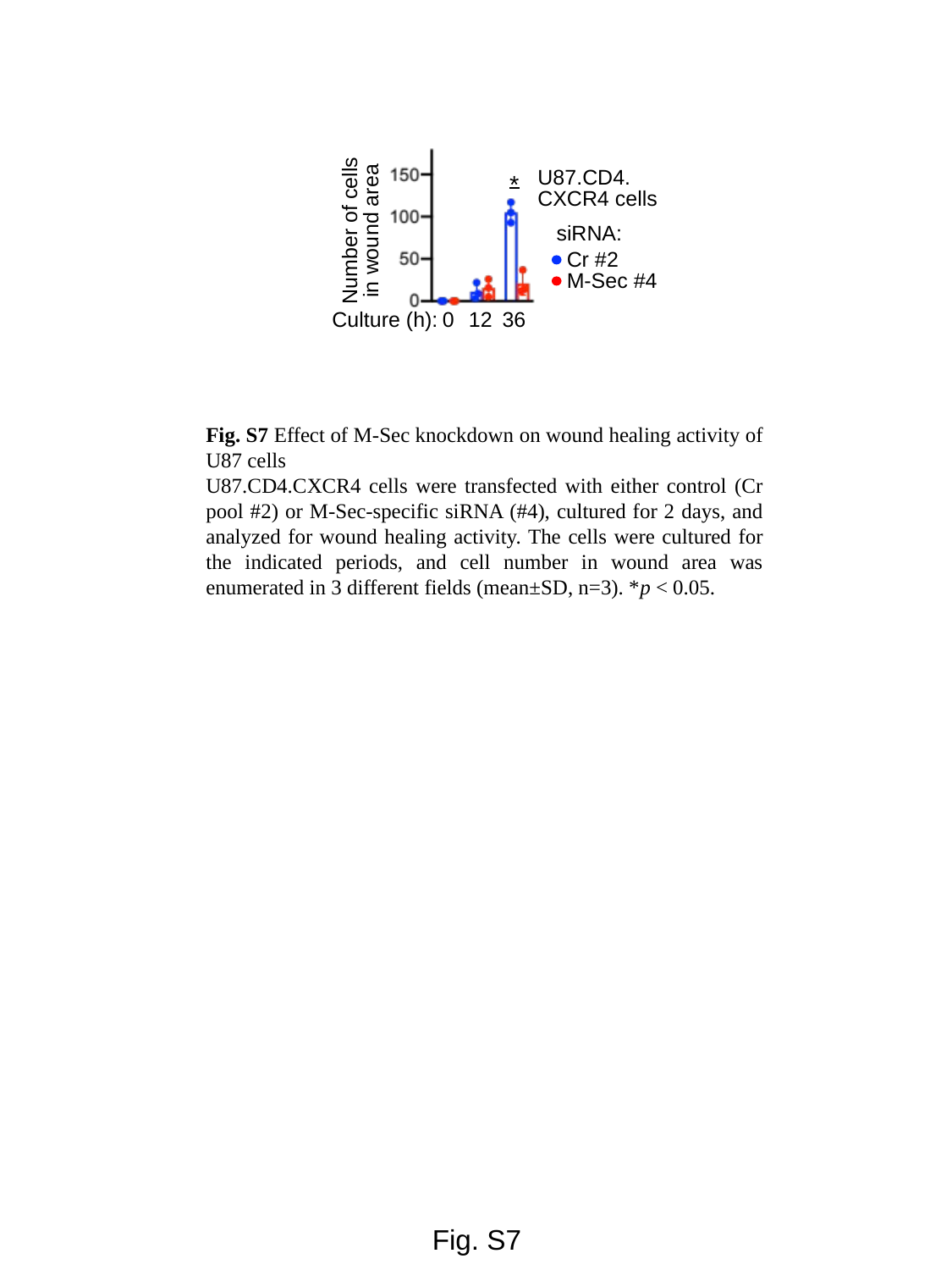

U87.CD4.
CXCR4 cells
*
Number of cells
in wound area
siRNA:
Cr #2
M-Sec #4
Culture (h):
0
12
36
Fig. S7 Effect of M-Sec knockdown on wound healing activity of U87 cells
U87.CD4.CXCR4 cells were transfected with either control (Cr pool #2) or M-Sec-specific siRNA (#4), cultured for 2 days, and analyzed for wound healing activity. The cells were cultured for the indicated periods, and cell number in wound area was enumerated in 3 different fields (mean±SD, n=3). *p < 0.05.
Fig. S7

## Slide 8
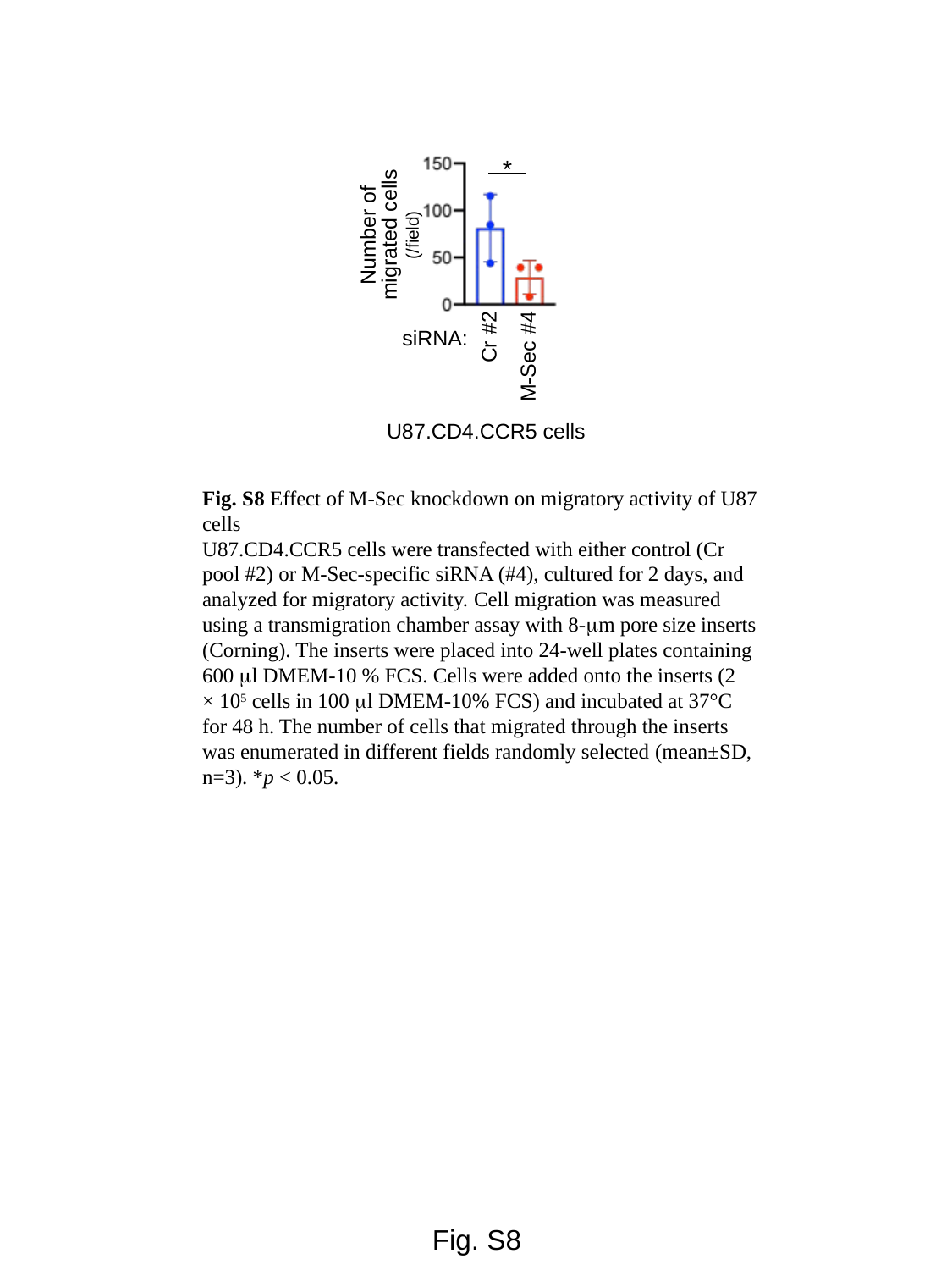

*
Number of
migrated cells
(/field)
Cr #2
siRNA:
M-Sec #4
U87.CD4.CCR5 cells
Fig. S8 Effect of M-Sec knockdown on migratory activity of U87 cells
U87.CD4.CCR5 cells were transfected with either control (Cr pool #2) or M-Sec-specific siRNA (#4), cultured for 2 days, and analyzed for migratory activity. Cell migration was measured using a transmigration chamber assay with 8-mm pore size inserts (Corning). The inserts were placed into 24-well plates containing 600 ml DMEM-10 % FCS. Cells were added onto the inserts (2
× 105 cells in 100 ml DMEM-10% FCS) and incubated at 37°C for 48 h. The number of cells that migrated through the inserts was enumerated in different fields randomly selected (mean±SD, n=3). *p < 0.05.
Fig. S8

## Slide 9
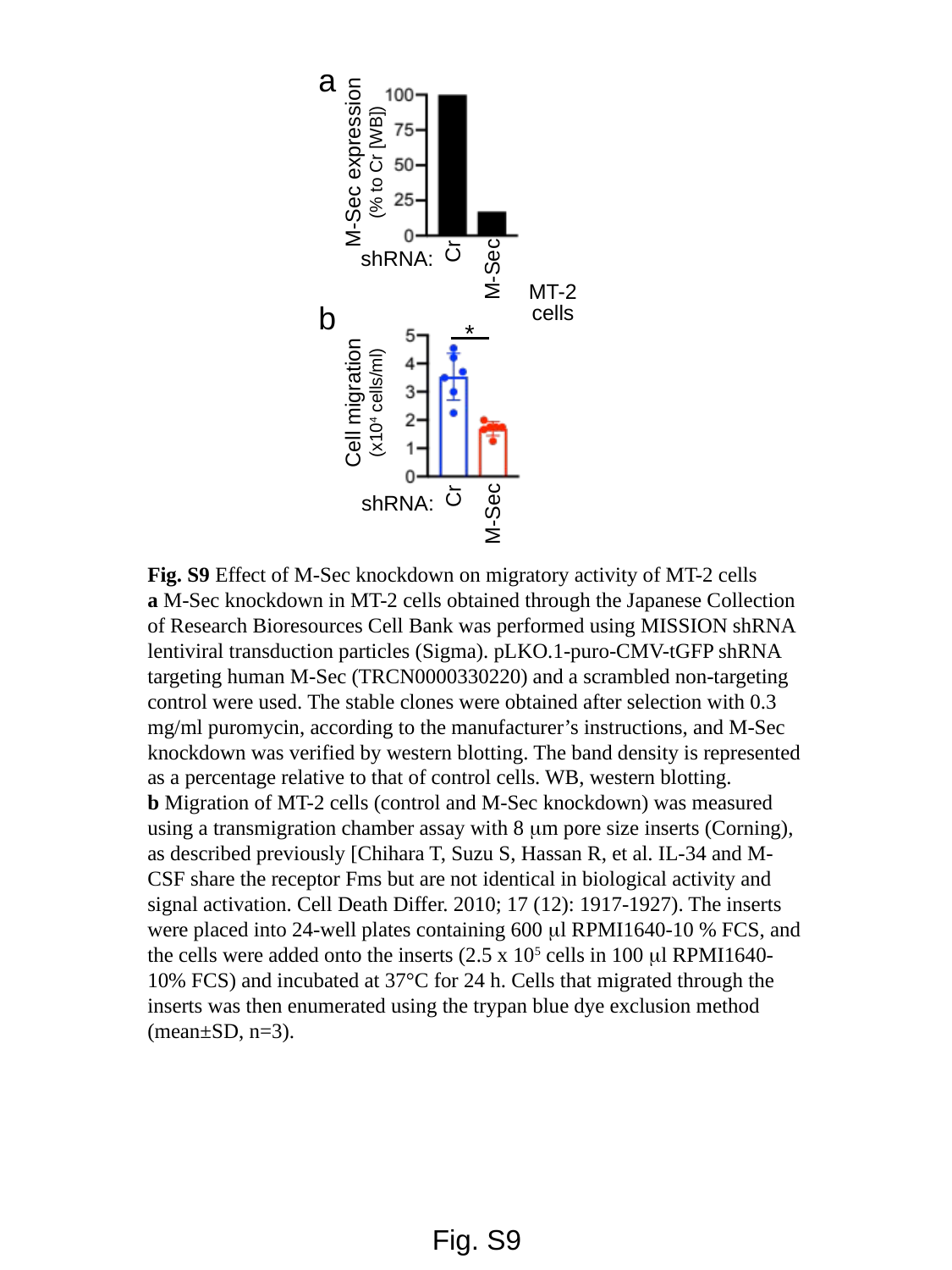

a
M-Sec expression
(% to Cr [WB])
Cr
shRNA:
M-Sec
MT-2
cells
b
*
Cell migration
(x104 cells/ml)
Cr
shRNA:
M-Sec
Fig. S9 Effect of M-Sec knockdown on migratory activity of MT-2 cells
a M-Sec knockdown in MT-2 cells obtained through the Japanese Collection of Research Bioresources Cell Bank was performed using MISSION shRNA lentiviral transduction particles (Sigma). pLKO.1-puro-CMV-tGFP shRNA targeting human M-Sec (TRCN0000330220) and a scrambled non-targeting control were used. The stable clones were obtained after selection with 0.3 mg/ml puromycin, according to the manufacturer’s instructions, and M-Sec knockdown was verified by western blotting. The band density is represented as a percentage relative to that of control cells. WB, western blotting.
b Migration of MT-2 cells (control and M-Sec knockdown) was measured using a transmigration chamber assay with 8 mm pore size inserts (Corning), as described previously [Chihara T, Suzu S, Hassan R, et al. IL-34 and M-CSF share the receptor Fms but are not identical in biological activity and signal activation. Cell Death Differ. 2010; 17 (12): 1917-1927). The inserts were placed into 24-well plates containing 600 ml RPMI1640-10 % FCS, and the cells were added onto the inserts (2.5 x 10^5^ cells in 100 ml RPMI1640-10% FCS) and incubated at 37°C for 24 h. Cells that migrated through the inserts was then enumerated using the trypan blue dye exclusion method (mean±SD, n=3).
Fig. S9

## Slide 10
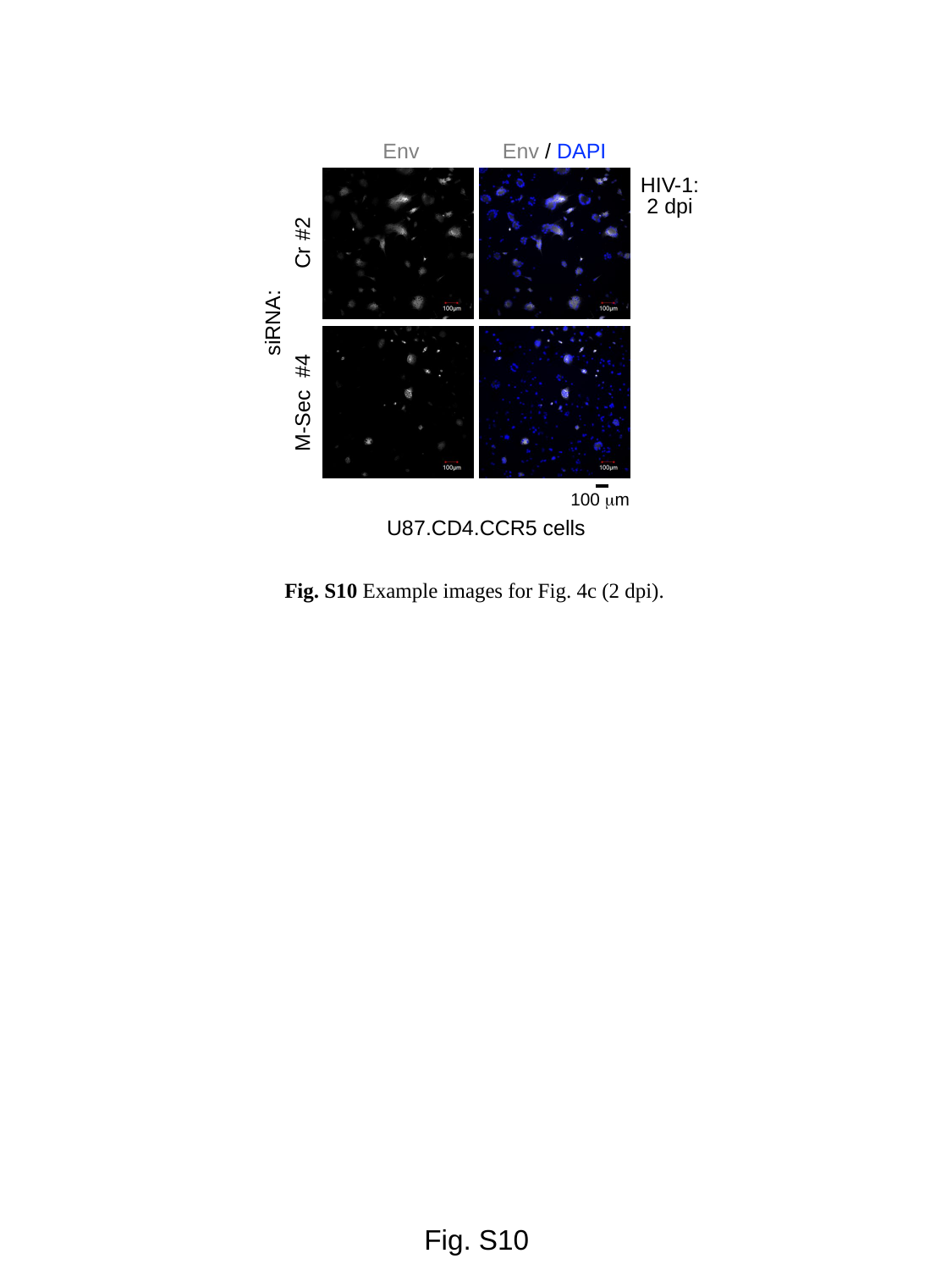

Env
Env / DAPI
HIV-1:
2 dpi
Cr #2
siRNA:
M-Sec #4
100 mm
U87.CD4.CCR5 cells
Fig. S10 Example images for Fig. 4c (2 dpi).
Fig. S10

## Slide 11
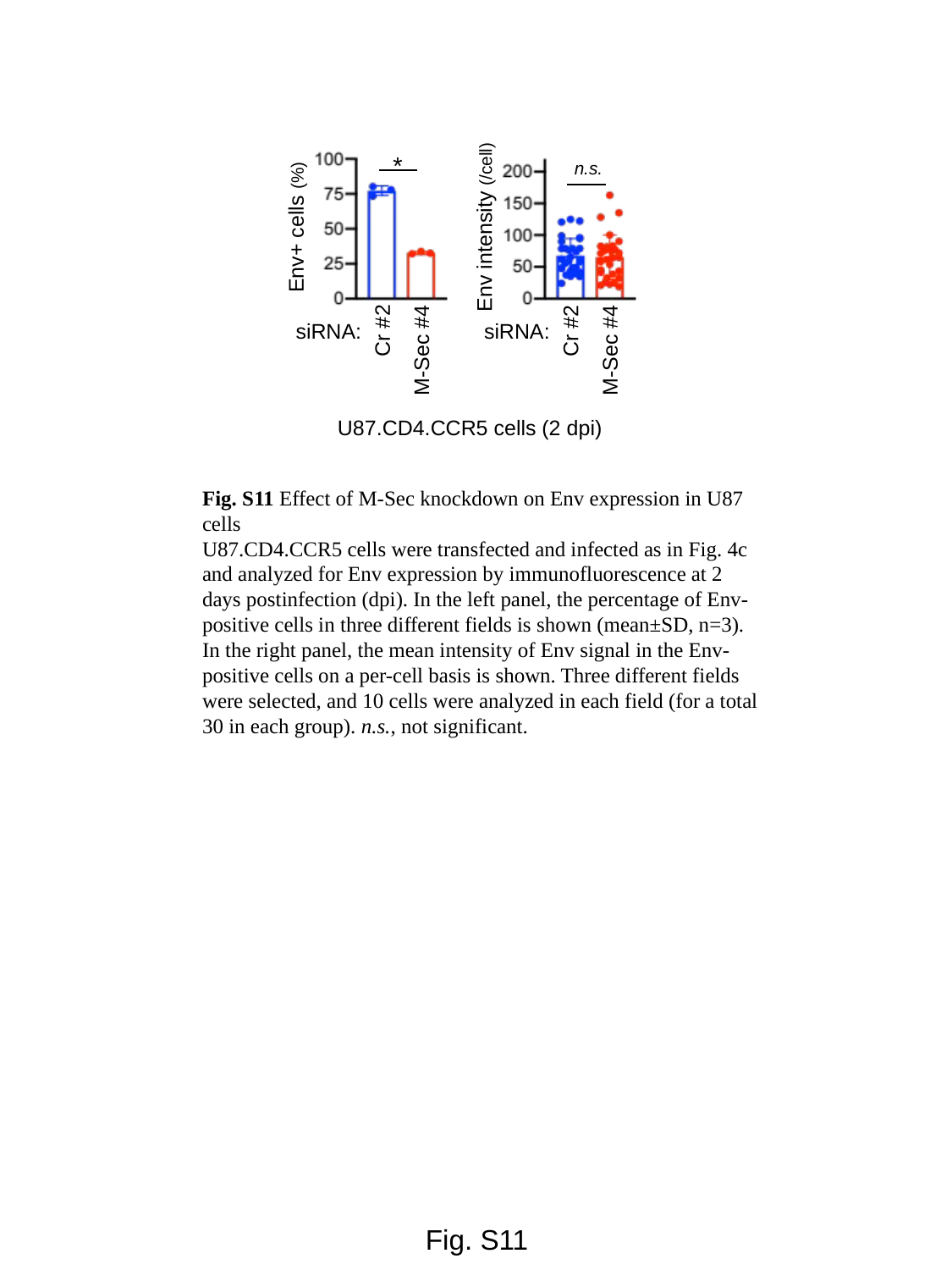

*
n.s.
Env+ cells (%)
Env intensity (/cell)
Cr #2
Cr #2
siRNA:
siRNA:
M-Sec #4
M-Sec #4
U87.CD4.CCR5 cells (2 dpi)
Fig. S11 Effect of M-Sec knockdown on Env expression in U87 cells
U87.CD4.CCR5 cells were transfected and infected as in Fig. 4c and analyzed for Env expression by immunofluorescence at 2 days postinfection (dpi). In the left panel, the percentage of Env-positive cells in three different fields is shown (mean±SD, n=3). In the right panel, the mean intensity of Env signal in the Env-positive cells on a per-cell basis is shown. Three different fields were selected, and 10 cells were analyzed in each field (for a total 30 in each group). n.s., not significant.
Fig. S11

## Slide 12
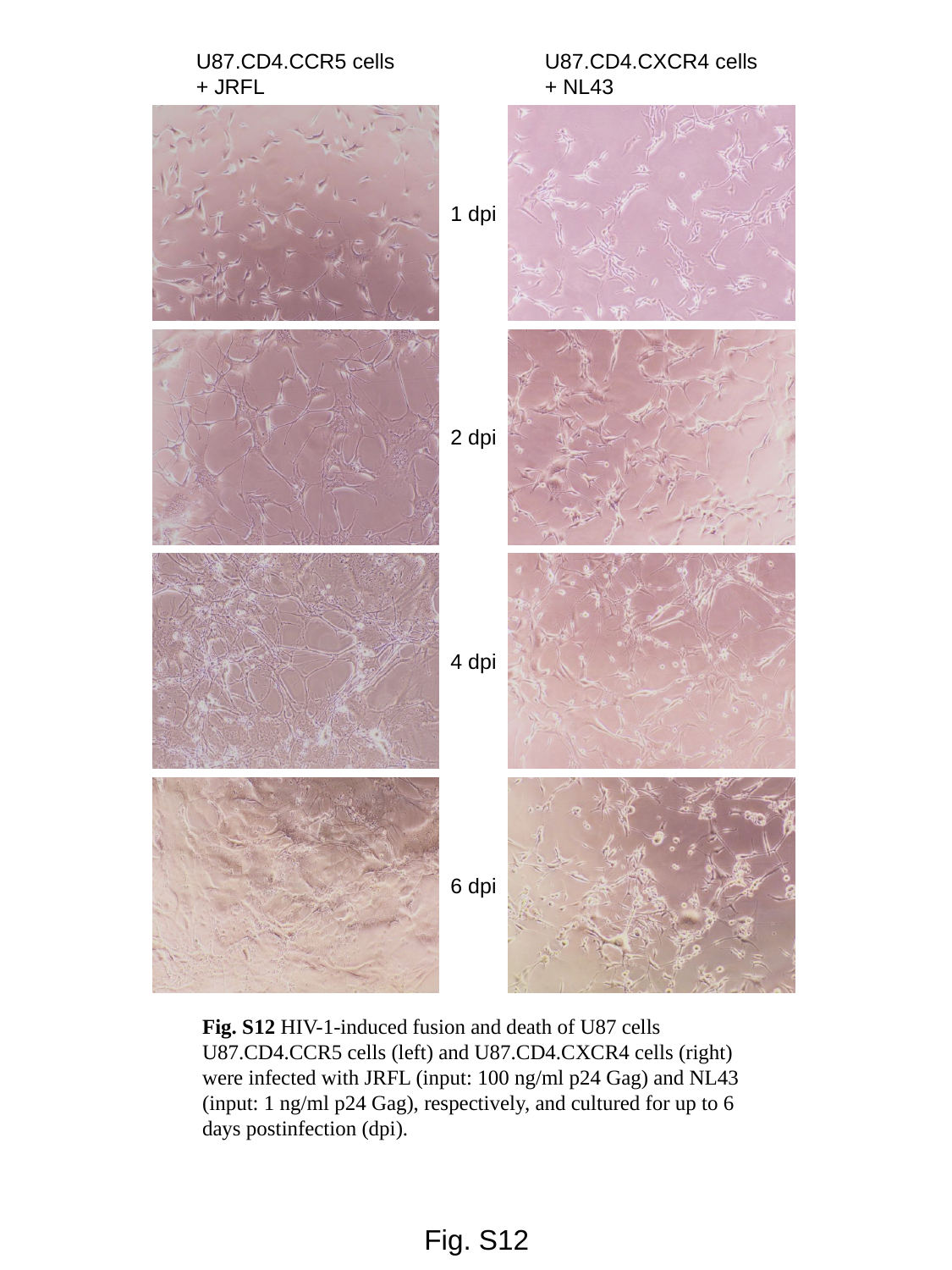

U87.CD4.CCR5 cells
+ JRFL
U87.CD4.CXCR4 cells
+ NL43
1 dpi
2 dpi
4 dpi
6 dpi
Fig. S12 HIV-1-induced fusion and death of U87 cells
U87.CD4.CCR5 cells (left) and U87.CD4.CXCR4 cells (right) were infected with JRFL (input: 100 ng/ml p24 Gag) and NL43 (input: 1 ng/ml p24 Gag), respectively, and cultured for up to 6 days postinfection (dpi).
Fig. S12

## Slide 13
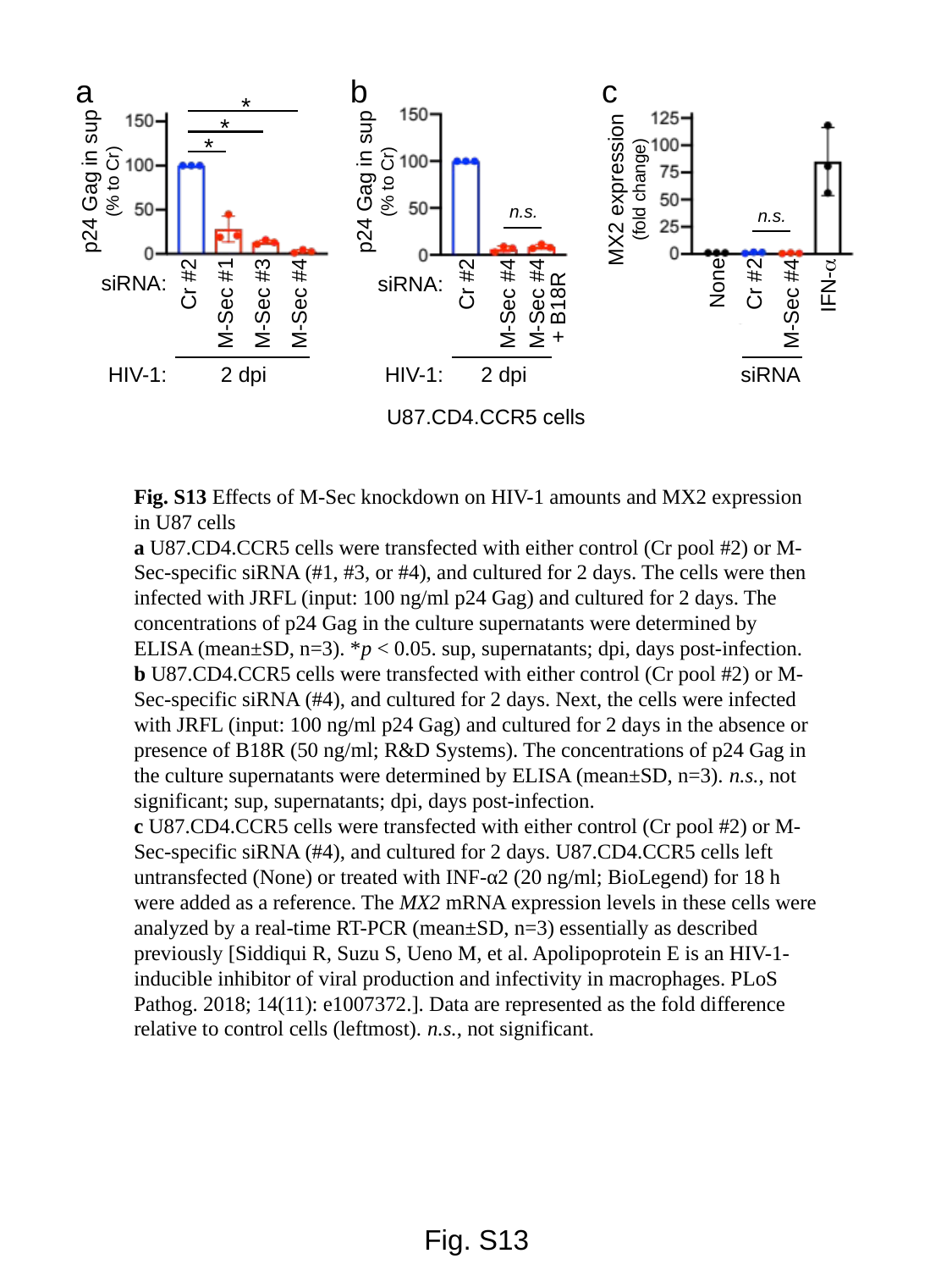

a
b
c
*
*
*
p24 Gag in sup
(% to Cr)
p24 Gag in sup
(% to Cr)
MX2 expression
(fold change)
n.s.
n.s.
siRNA:
siRNA:
None
Cr #2
Cr #2
Cr #2
IFN-a
M-Sec #4
 + B18R
M-Sec #1
M-Sec #3
M-Sec #4
M-Sec #4
M-Sec #4
HIV-1:
2 dpi
siRNA
HIV-1:
2 dpi
U87.CD4.CCR5 cells
Fig. S13 Effects of M-Sec knockdown on HIV-1 amounts and MX2 expression in U87 cells
a U87.CD4.CCR5 cells were transfected with either control (Cr pool #2) or M-Sec-specific siRNA (#1, #3, or #4), and cultured for 2 days. The cells were then infected with JRFL (input: 100 ng/ml p24 Gag) and cultured for 2 days. The concentrations of p24 Gag in the culture supernatants were determined by ELISA (mean±SD, n=3). *p < 0.05. sup, supernatants; dpi, days post-infection.
b U87.CD4.CCR5 cells were transfected with either control (Cr pool #2) or M-Sec-specific siRNA (#4), and cultured for 2 days. Next, the cells were infected with JRFL (input: 100 ng/ml p24 Gag) and cultured for 2 days in the absence or presence of B18R (50 ng/ml; R&D Systems). The concentrations of p24 Gag in the culture supernatants were determined by ELISA (mean±SD, n=3). n.s., not significant; sup, supernatants; dpi, days post-infection.
c U87.CD4.CCR5 cells were transfected with either control (Cr pool #2) or M-Sec-specific siRNA (#4), and cultured for 2 days. U87.CD4.CCR5 cells left untransfected (None) or treated with INF-α2 (20 ng/ml; BioLegend) for 18 h were added as a reference. The MX2 mRNA expression levels in these cells were analyzed by a real-time RT-PCR (mean±SD, n=3) essentially as described previously [Siddiqui R, Suzu S, Ueno M, et al. Apolipoprotein E is an HIV-1-inducible inhibitor of viral production and infectivity in macrophages. PLoS Pathog. 2018; 14(11): e1007372.]. Data are represented as the fold difference relative to control cells (leftmost). n.s., not significant.
Fig. S13

## Slide 14
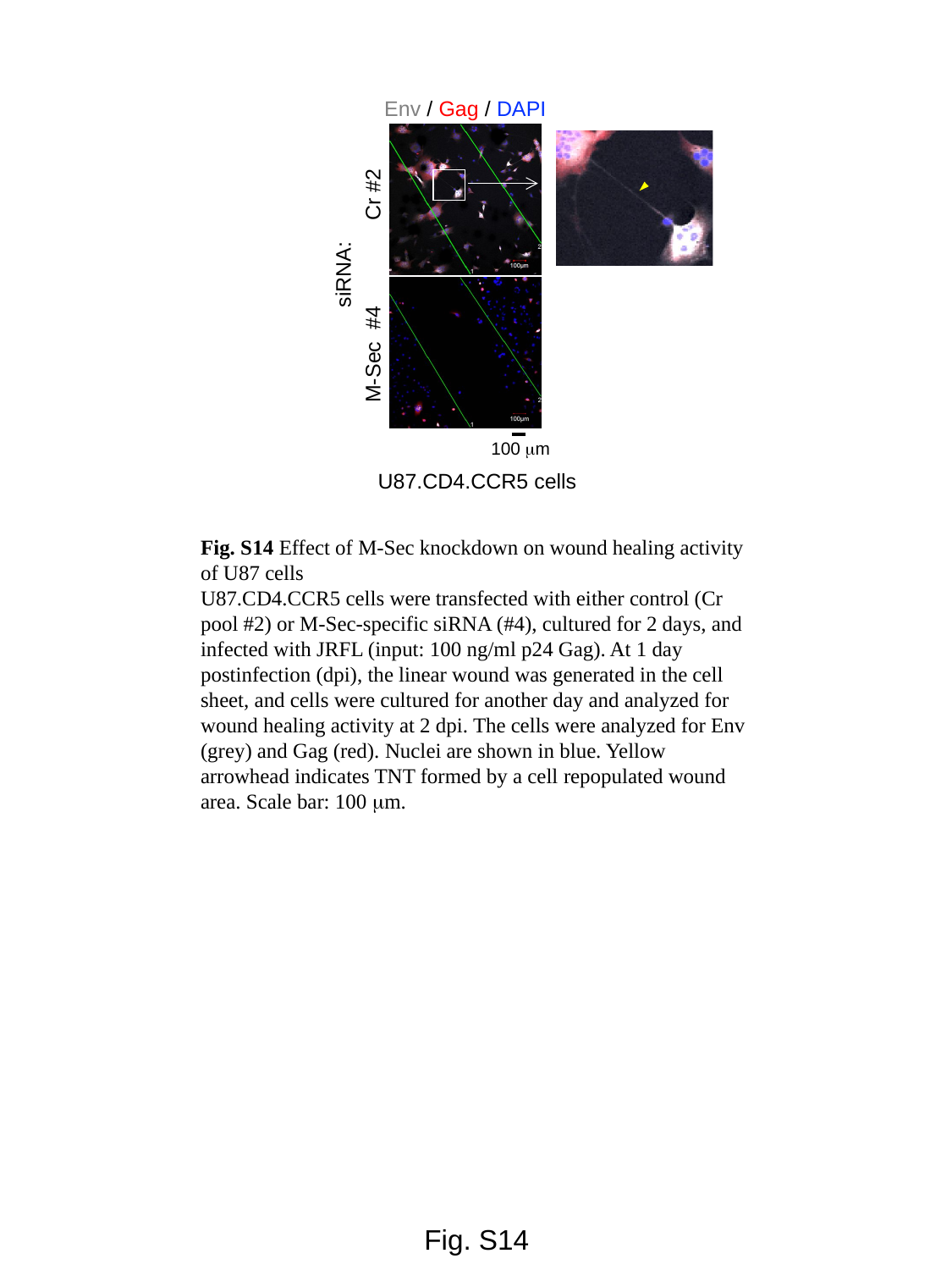

Env / Gag / DAPI
Cr #2
siRNA:
M-Sec #4
100 mm
U87.CD4.CCR5 cells
Fig. S14 Effect of M-Sec knockdown on wound healing activity of U87 cells
U87.CD4.CCR5 cells were transfected with either control (Cr pool #2) or M-Sec-specific siRNA (#4), cultured for 2 days, and infected with JRFL (input: 100 ng/ml p24 Gag). At 1 day postinfection (dpi), the linear wound was generated in the cell sheet, and cells were cultured for another day and analyzed for wound healing activity at 2 dpi. The cells were analyzed for Env (grey) and Gag (red). Nuclei are shown in blue. Yellow arrowhead indicates TNT formed by a cell repopulated wound area. Scale bar: 100 mm.
Fig. S14

## Slide 15
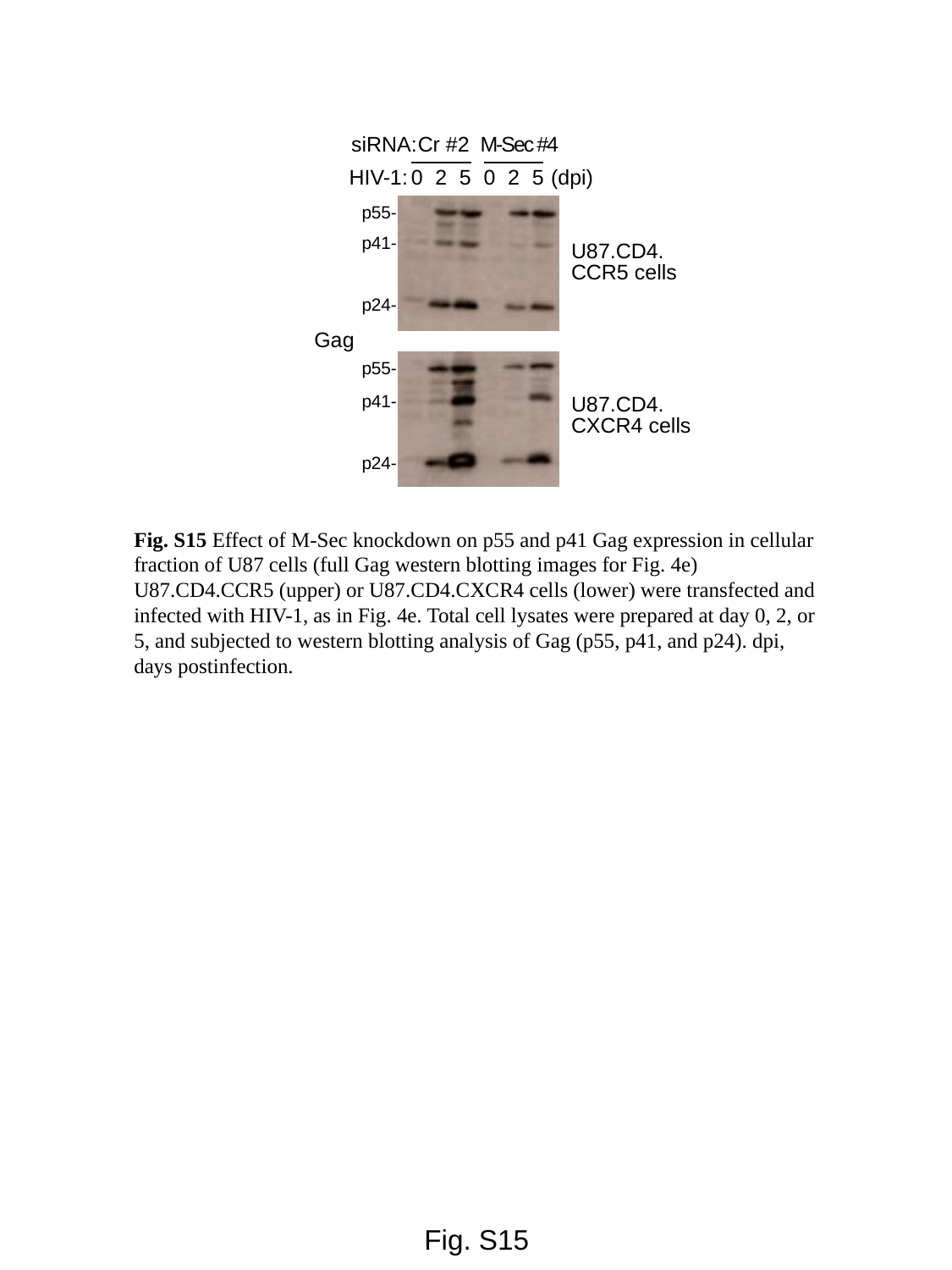

siRNA:
Cr #2
M-Sec #4
HIV-1:
0
2
5
0
2
5
(dpi)
p55-
p41-
U87.CD4.
CCR5 cells
p24-
Gag
p55-
p41-
U87.CD4.
CXCR4 cells
p24-
Fig. S15 Effect of M-Sec knockdown on p55 and p41 Gag expression in cellular fraction of U87 cells (full Gag western blotting images for Fig. 4e)
U87.CD4.CCR5 (upper) or U87.CD4.CXCR4 cells (lower) were transfected and infected with HIV-1, as in Fig. 4e. Total cell lysates were prepared at day 0, 2, or 5, and subjected to western blotting analysis of Gag (p55, p41, and p24). dpi, days postinfection.
Fig. S15

## Slide 16
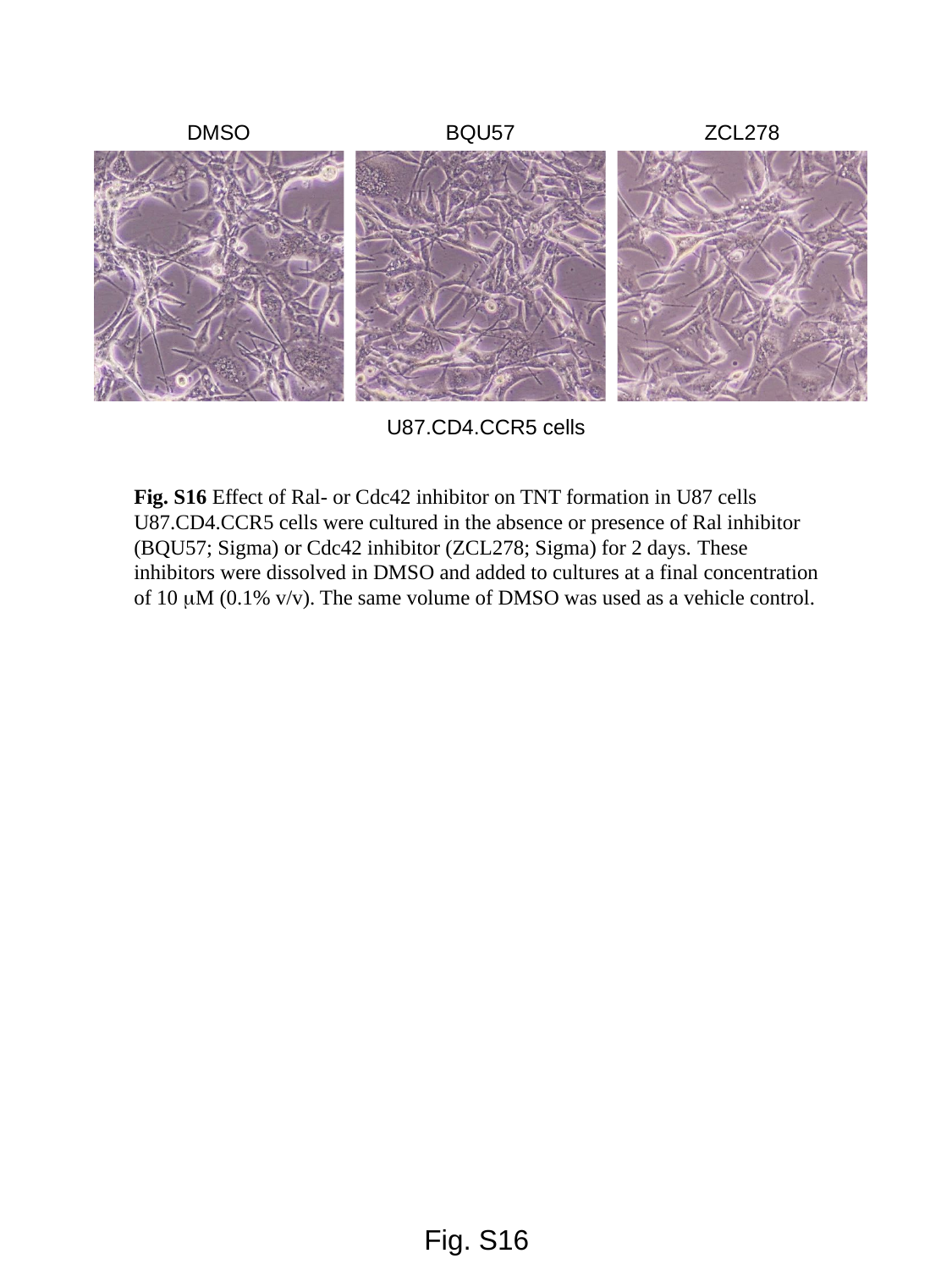

DMSO
BQU57
ZCL278
U87.CD4.CCR5 cells
Fig. S16 Effect of Ral- or Cdc42 inhibitor on TNT formation in U87 cells
U87.CD4.CCR5 cells were cultured in the absence or presence of Ral inhibitor (BQU57; Sigma) or Cdc42 inhibitor (ZCL278; Sigma) for 2 days. These inhibitors were dissolved in DMSO and added to cultures at a final concentration of 10 mM (0.1% v/v). The same volume of DMSO was used as a vehicle control.
Fig. S16
